# Publisher Correction: Thermodiffusive desalination

**DOI:** 10.1038/s41467-024-48716-0

**Published:** 2024-05-17

**Authors:** Shuqi Xu, Alice J. Hutchinson, Mahdiar Taheri, Ben Corry, Juan F. Torres

**Affiliations:** 1 ANU HEAT Lab, School of Engineering, The Australian National University, Canberra, ACT Australia; 2 Research School of Biology, The Australian National University, Canberra, ACT Australia

**Keywords:** Sustainability, Water resources, Applied physics, Mechanical engineering, Chemical physics

Correction to: *Nature Communications* 10.1038/s41467-024-47313-5, published online 08 April 2024

The original version of this Article contained an error in the citation of reference [19] which was incorrectly given as “Soret, C. Sur l’ćtat d’ćquilibre que prend, au point de vue de sa concentration, une dissolution saline primitivement homogc`ne, dont deux parties sont portćes à des tempćratures diffćrentes; Archives de Genc`ve, 3e Periode, t. II, p. 48; 1879. J. Phys. Theor. Appl. 9, 331–332 (1879).” The correct citation is “Soret, C. Sur l’état d’équilibre Que Prend, Au Point de Vue de Sa Concentration, Une Dissolution Saline Primitivement Homogène, Dont Deux Parties Sont Portées à Des Températures Différentes; Archives de Genève, 3e Periode. J. Phys. Theor. Appl. 9, 331–332 (1879).” This has been corrected in the PDF and HTML versions of the Article.

