# Supplementary material for: Thermodiffusive desalination
Source: Nat Commun. 2024 Apr 8;15:2996. doi: 10.1038/s41467-024-47313-5 (PMC10999432; doi:10.1038/s41467-024-47313-5)
Supplement: Supplementary file 1 — Supplementary Information [file 41467_2024_47313_MOESM1_ESM.pdf]

Supplementary Information

Thermodiffusive desalination

Shuqi Xu,[1] *Alice J. Hutchinson,*[1,2] *Mahdiar Taheri,*[1] *Ben Corry*[2] *and Juan F. Torres*[1]

[1]ANU HEAT Lab, School of Engineering, Australian National University, Canberra, Australia

[2]Research School of Biology, Australian National University, Canberra, Australia

Supplementary Figures

Fig. 1 Photo of the thermodiffusive desalination unit (TDU) 2
Fig. 2 Thermodiffusive transport models based on continuum mechanics 3
Fig. 3 Verification of continuum thermodiffusion model 3
Fig. 4 Validation of continuum thermodiffusion model 4
Fig. 5 Effect of different parameters on thermodiffusive separation 5
Fig. 6 TDU design considerations 6
Fig. 7 Modelled concentration difference along the channel for various temperature controls . 6
Fig. 8 Highly-accurate measurement of concentration difference with interferometry 7
Fig. 9 MD simulation: convergence of NaCl brine concentration profile. 8
Fig. 10 MD simulation: convergence of seawater brine concentration profile. 9
Fig. 11 MD simulation: concentration profiles of individual ions in modelled brine. 10
Fig. 12 Burgers cascade: a single flow pass multi-channel device 11
Fig. 13 Burgers cascade: pressure drop, yield and heat flux 12
Fig. 14 Theoretical minimum energy of separation for desalination 12

Supplementary Tables

Table 1 Ion composition of binary and multi-ion brine solutions modelled in MD simulations. 13
Table 2 Linear fitting parameters and Soret coefficients from multi-pass experiments 14
Table 3 Comparison of thermodiffusive desalination with other emerging technologies. 15
Table 4 Reference list for desalination technology comparison (used in Fig. 6) 34

Supplementary Methods

Method 1 Modelling of continuum thermodiffusion 16
Method 2 Design rationale for a thermodiffusive desalination channel 19
Method 3 Measurement of thermodiffusive separation 22
Method 4 Temperature profile in separation channel 24
Method 5 Multi-component saline water re-circulation 24
Method 6 Molecular dynamics modelling 27
Method 7 Burgers cascade modelling 31
Method 8 Energy consumption and thermodynamic limit 31

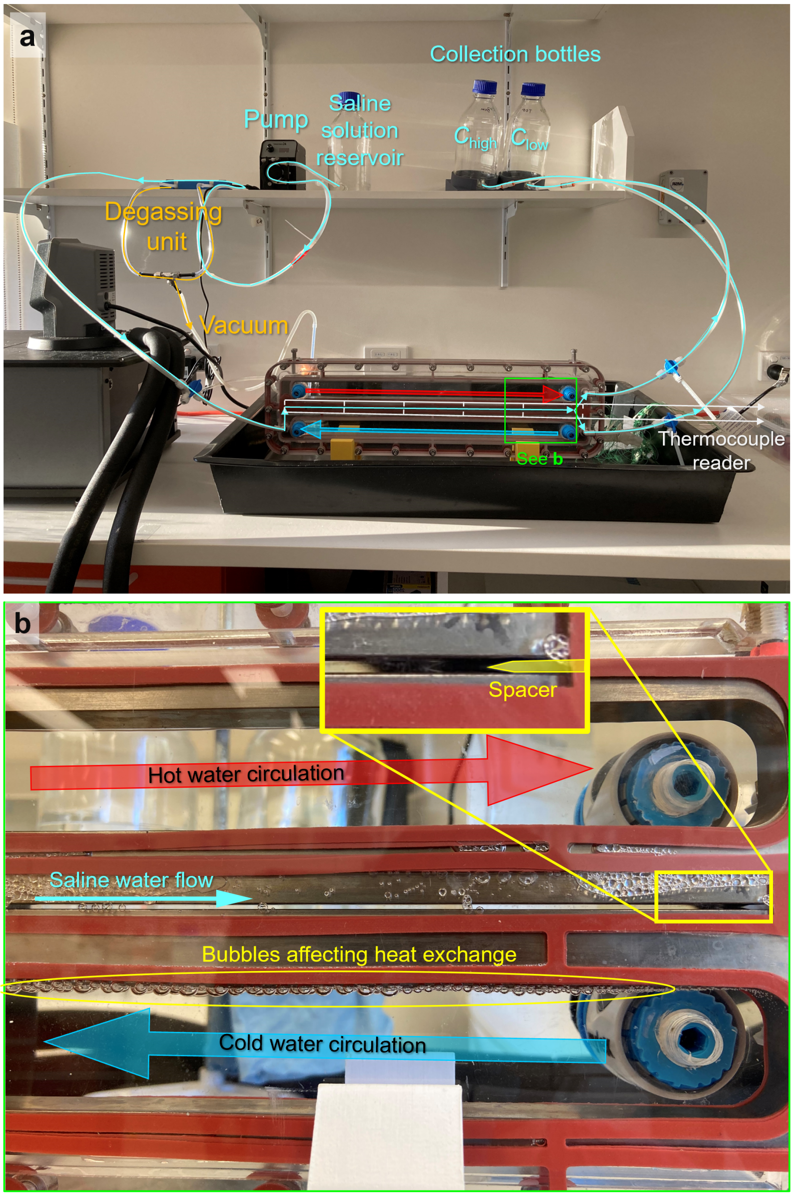

Supplementary Figure 1 | Photo of the thermodiffusive desalination unit (TDU). a, The concept and unit design is shown in Fig. 1. Different parts are labelled. The saline water flow is indicated by cyan lines. At the degassing unit, the saline water flow and the vacuum (bright orange line) is separated by polydimethylsiloxane (PDMS) membrane which allows gas exchange. There are six pairs of thermocouples (white lines) distributed at 100 mm interval alone the 500 mm-long TDU channel. The channel is made by firmly sandwiching the spacer with two nickel-plated copper blocks from the top and the bottom. Then compressing with acrylic plates from the sides to confine the liquid. The acrylic plates allow direct visual inspection of the flow through the channel. The thick red and blue arrows indicate the direction of the water circulation within the copper blocks used for the temperature control, which in this case is a counter-flow configuration (temperature reported in Supplementary Fig. 7b). **b,** Magnified image of the saline water channel, from green region in **a**. Saline water flow is indicated by the bright blue arrow. The channel is bifurcated by a spacer at the outlet, which is highlighted by the bright yellow region in the inset. The bubbles that accumulate on the top surface of the cold water circulation chamber are indicated. This is a factor affecting the temperature profile along the channel, thus the repeatability of the experiment.

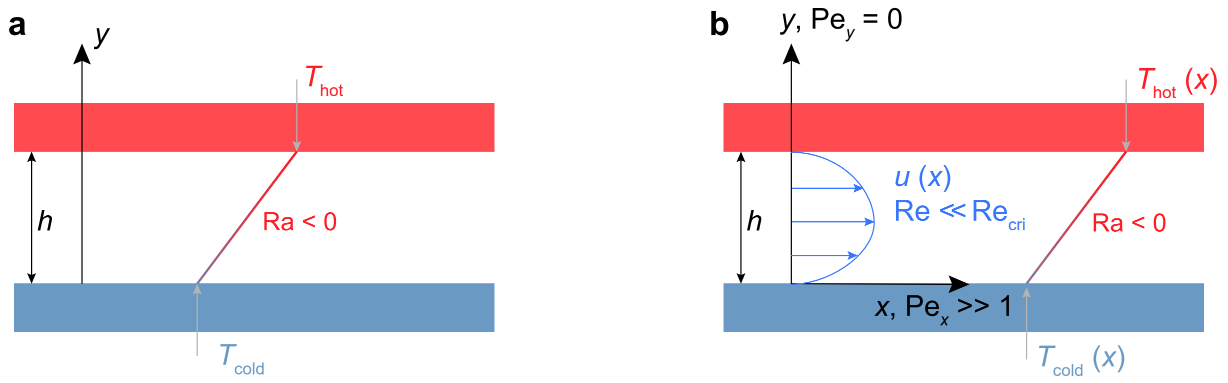

Supplementary Figure 2 | Thermodiffusive transport models based on continuum mechanics. A positive temperature gradient is present in the y (vertical) direction within the rectangular channel. **a,** Convectionless model. The fluid is stationary. One-dimensional thermodiffusion occurs in the y direction due to a quasi-linear temperature profile. The top wall is hot at a constant temperature of T_{hot} and the bottom wall is cold at T_{cold}. In steady state, a linear temperature profile between top and bottom walls can be assumed because the thermal conductivity is nearly temperature-independent. **b,** Convection model. Two-dimensional thermodiffusion in a fully-developed plane Poiseuille flow. Fluid flow occurs in the x direction. The top wall is hot at a temperature of $T_{\text{hot}}(x)$ and the bottom wall is cold at a temperature of $T_{\text{cold}}(x)$. A quasi-linear temperature profile exists between the top and bottom walls because the flow has a low Reynolds number Re. Through analysis (Supplementary Method 2), it was found that the flow in the parallel-plate channel is well within the laminar flow regime and both the thermal and hydrodynamic entrance length are much smaller than the total channel length. In the x direction, the mass and heat transfer are advection-dominant while in the y direction they are diffusion-dominant.

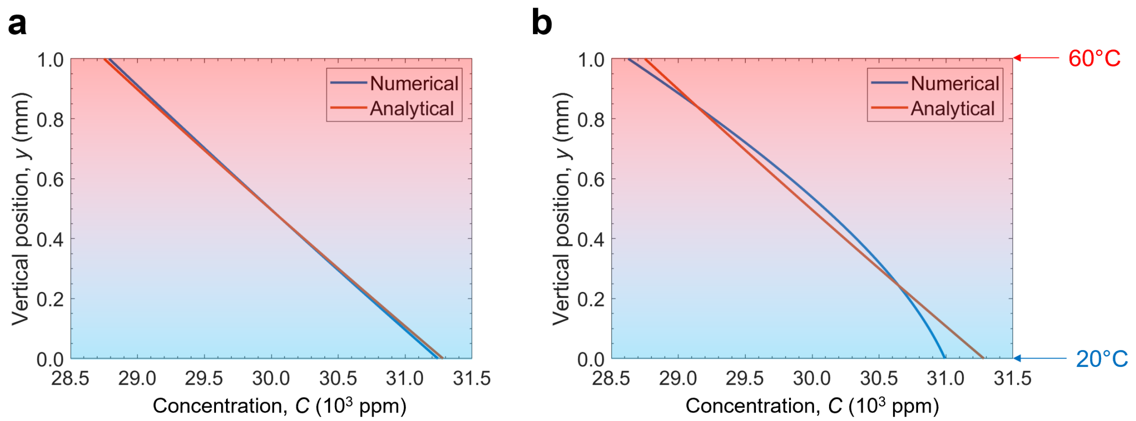

Supplementary Figure 3 | Verification of continuum thermodiffusion model. The concentration profile is the steady-state concentration profile in the absence of a fluid flow for the aqueous NaCl solution at $T_{\text{mean}} = 40\,°\text{C}$ and $\Delta T = 40\,\text{K}$. **a,** The values of D and D_{T} are assumed constant and evaluated at the mean temperature T_{mean}. In this case, the concentration profile is linear under linear temperature gradient. A good agreement is observed between numerical results and the analytical solution. **b,** The coefficients D and D_{T} are now considered as temperature dependent in the simulation. In the region where the local temperature is high, the concentration gradient $\frac{\partial C}{\partial y}$ is larger because thermodiffusion is stronger, i.e. S_{T} increases with temperature.

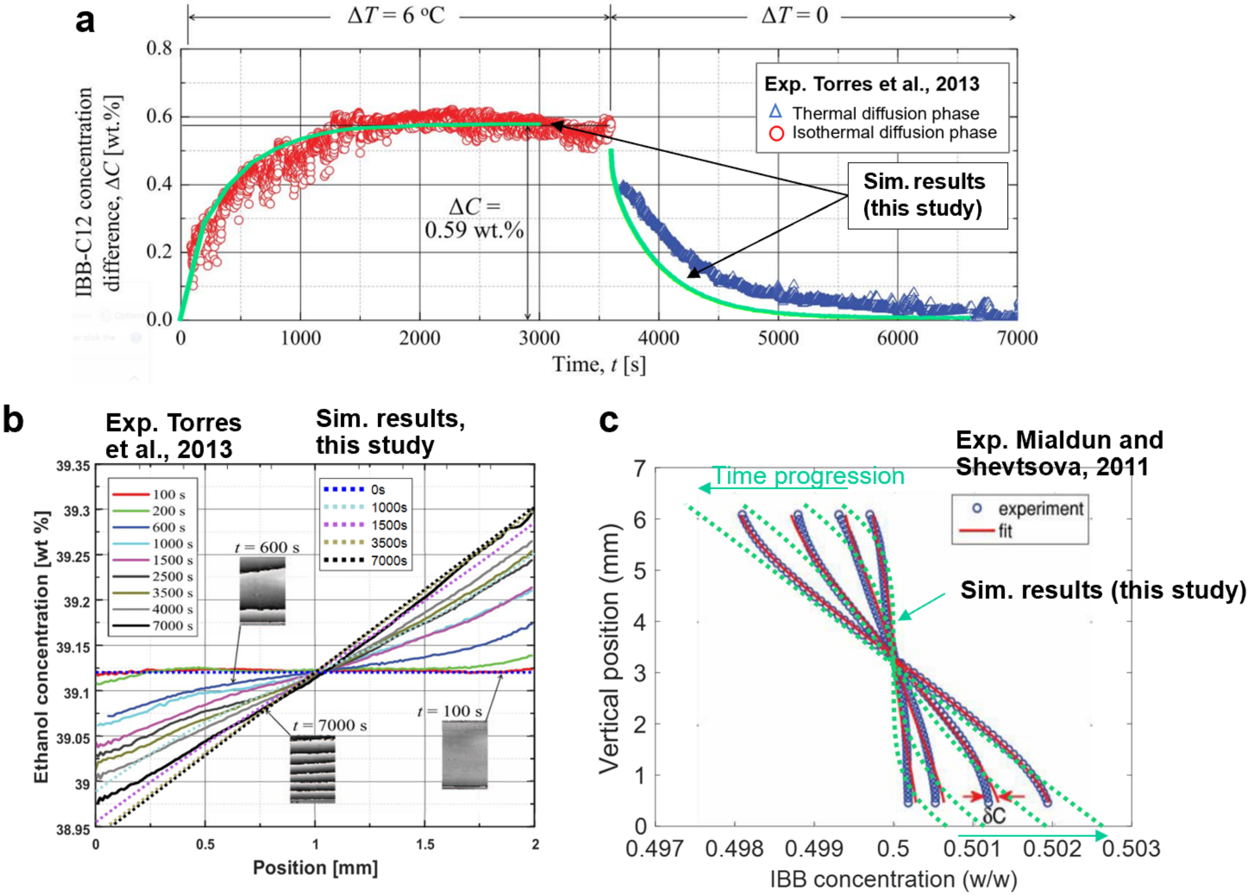

Supplementary Figure 4 | Validation of continuum thermodiffusion model. Comparison of experimental data in the literature and our simulation results for transient thermodiffusion without advection. **a,** Experimental data from phase-shifting interferometry for an isobutylbenzene (IBB) and dodecane (C_{12}) binary solution with equal weight fractions [1]. **b,** Ethanol–water with an ethanol weight fraction of 0.3912 and $\Delta T = 5\,K$ [1]. **c,** IBB-$C_{12}H_{26}$ with equal weight fraction, $\Delta T = 5.53\,K$, $T_{\mathrm{mean}} = 25\,°C$, and Soret cell height of 6.3 mm [2].

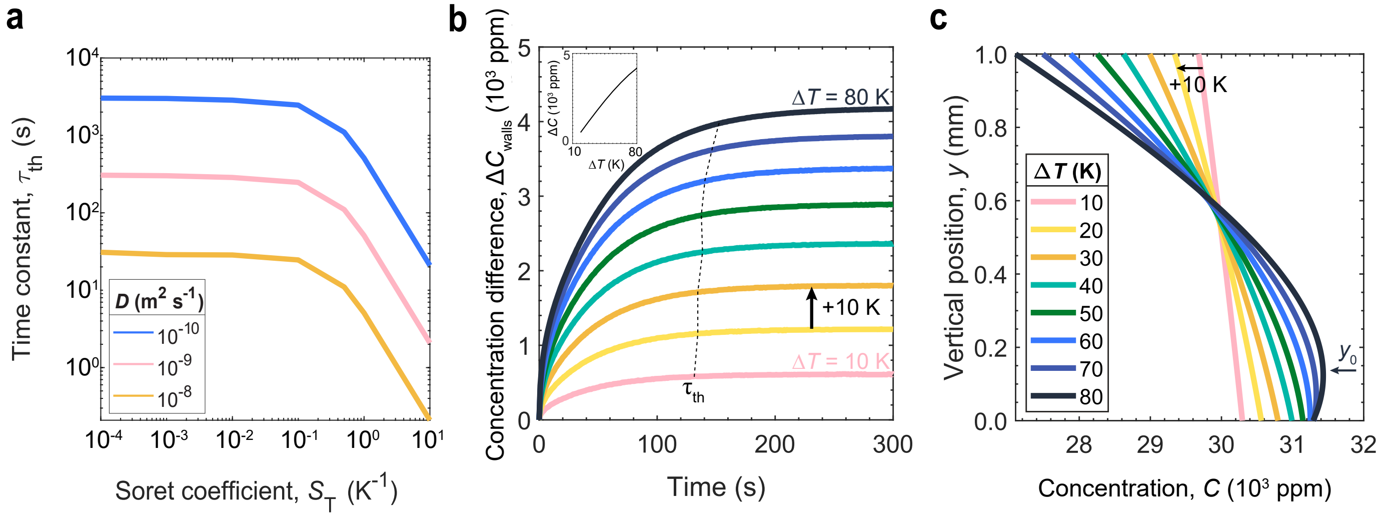

Supplementary Figure 5 | Effect of different parameters on thermodiffusive separation. a, The effect of the D and S_T on the thermodiffusion time constant τ_{th}, i.e. the time required for the concentration profile to reach steady state. For most species where $S_T < 0.1\,K^{-1}$ (includes seawater), τ_{th} is only dependent on the isothermal diffusion coefficient D. Moreover, our simulation results show that $\tau_{th} \propto D^{-1}$, agreeing with Eq. (8). In contrast, for $S_T > 0.1\,K^{-1}$, the thermodiffusion process starts to exhibit an effect on the time response as per observed τ_{th} dependence on S_T. **b,** Transient thermodiffusive separation for a convectionless aqueous NaCl solution of initial concentration $C_0 = 30\,000$ ppm when $T_{mean} = 40\,°C$. ΔC_{walls} between boundaries is plotted as a function of time. In steady state, the maximum separation ΔC_{steady} is achieved. For the same T_{mean}, the time taken (indicated by dashed line) to reach steady state is roughly the same despite different ΔT. In the inset, the ΔC_{walls} at τ_{th} is plotted against ΔT. The relationship is nearly linear particularly when ΔT is smaller than 60 K. **c,** An aqueous NaCl solution with the same C_0 passes through a parallel-plate channel with a fully-developed parabolic velocity profile. The vertical steady-state concentration profile is plotted for different ΔT at $T_{mean} = 40\,°C$ (note that we analyse the extreme case where the local temperature is close to 0 °C). When the inversion temperature $T_0 = 12\,°C$ is within the set temperature range, NaCl is thermophobic in the hotter region and thermophilic in the colder region, yielding a concentration peak at y_0 corresponding to the location of T_0, e.g. $y_0 = 143\,\mu m$ for $\Delta T = 80\,K$.

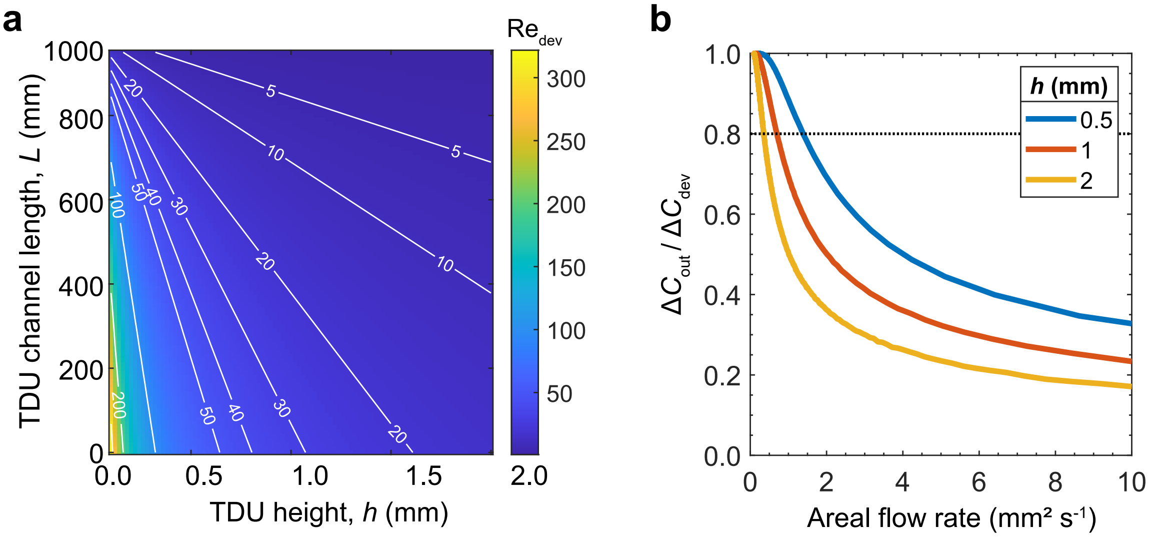

Supplementary Figure 6 | TDU design considerations. a, Reynolds number, Re, as a function of the TDU channel length and height when the flow speed of the NaCl/H_2O fluid in the channel is limited by the thermodiffusion time constant, τ_{th}. **b,** The separation ratio defined as the ratio of concentration difference between hot and cold walls at the channel outlet to that of the maximum possible separation in a fully-developed condition, i.e. $\Delta C_{\text{out}}/\Delta C_{\text{dev}}$, is plotted as a function of the areal flow rate, i.e. volumetric flow rate per unit channel width.

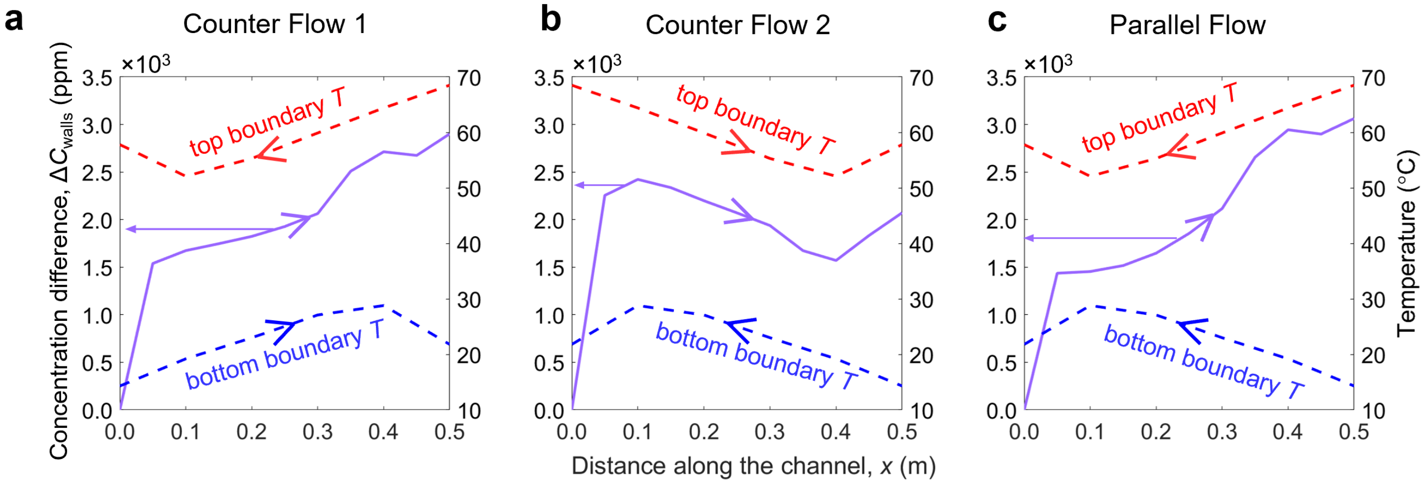

Supplementary Figure 7 | Modelled concentration difference along the channel for various temperature controls. The modelled ΔC_{walls} (solid line; left axis) and the measured wall temperatures (dashed lines; right axis) are plotted as a function of the distance along the channel. Water baths with different flow configuration are tested. The red and blue arrows represents the flow direction of hot and cold water baths, respectively. The saline water flow is from left to right. The volumetric flow rate is $1\,\text{mL}\,\text{min}^{-1}$, which is much smaller than the actual flow rate used in experiments. **a,** A counter-flow configuration where the hot water bath is in the opposite direction to the saline water flow. **b,** A counter-flow configuration where the hot water bath is in the same direction to the saline water flow. **c,** A parallel-flow configuration where both water baths are in the direction opposite to the saline water flow.

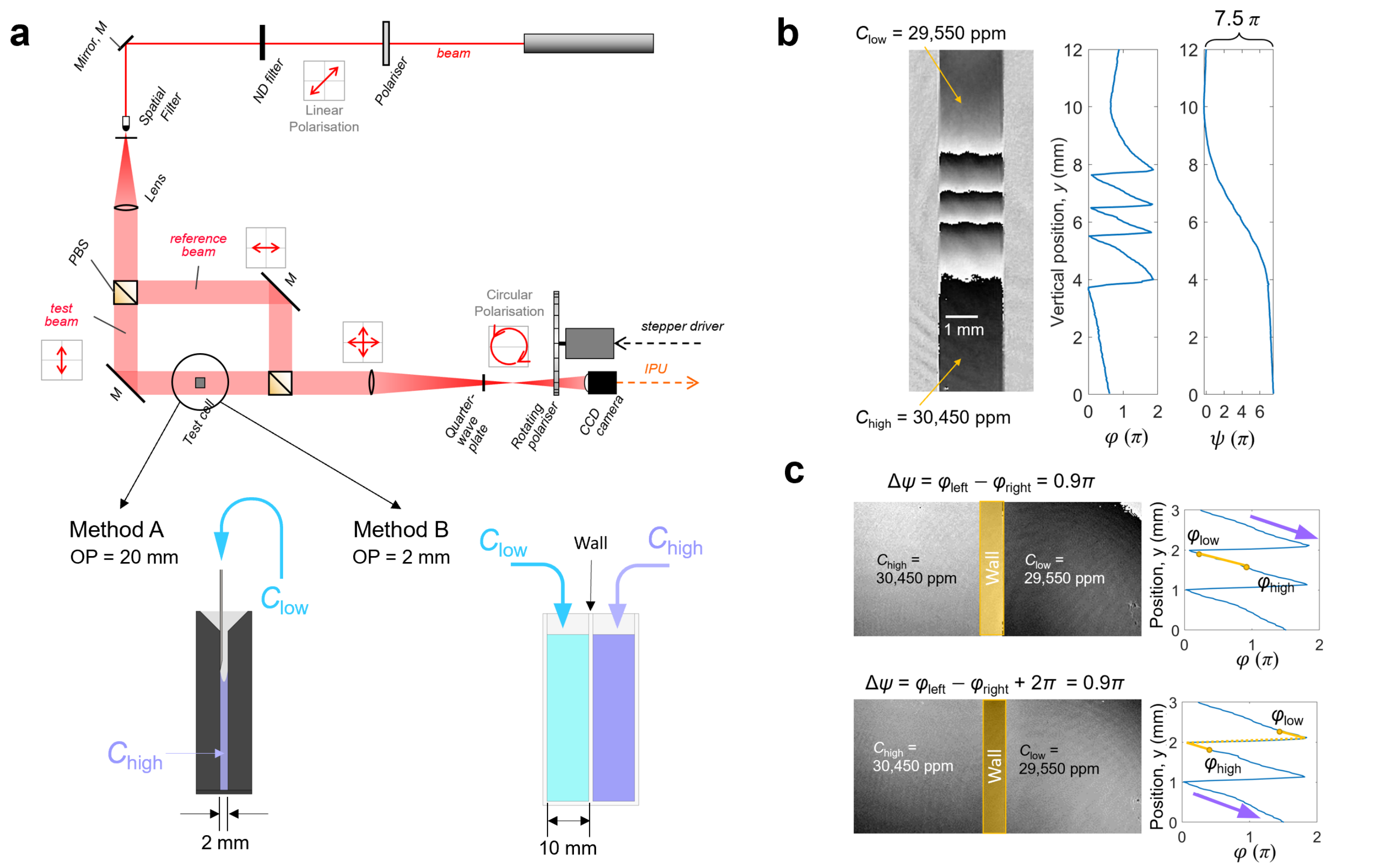

Supplementary Figure 8 | Highly-accurate measurement of concentration difference with interferometry. **a,** Layout of the polarising Mach–Zehnder interferometer, which is the basis for PSI, as described in [3]. Adapted from [3] with permission of Elsevier. The insets show the front view of test cells used in each measurement method of concentration difference. **b,** Method A: the contrast factor CF is measured by smoothly injecting a low-concentration solution on top of a high-concentration solution. On the left is the phase shifted image from PSI. The phase ϕ and the unwrapped phase ψ are plotted against the cell height on the right. Since $\psi = 7.5\pi$ rad for $\Delta C = 900$ ppm with an optical path of 20 mm, $\mathrm{CF} = \frac{\Delta\psi}{\mathrm{OP}\times\Delta C} = 0.42\pi\ \mathrm{g\,mg^{-1}\,mm}$. **c,** Method B: to ensure there was no mixing during injection, the two solutions are placed into a two-chamber quartz cell with an optical path of 2 mm. The phase-shifted image from PSI and the wrapped $\psi(y)$ plot are shown. In the $\psi(y)$ plot, the blue arrow indicates the direction where ψ is increasing. When the two solutions are placed in separate chambers, we are only able to observe two discrete points in the $\psi(y)$ plot but the number of fringes in between the two points cannot be seen. Thus a smaller optical path of 2 mm was chosen to makes sure that the $\psi_{\mathrm{high}} - \psi_{\mathrm{low}}$ is less than 2π. Moreover, $\psi_{\mathrm{high}} - \psi_{\mathrm{low}} > 0$ as CF is positive. When $\phi_{\mathrm{high}} - \phi_{\mathrm{low}} < 0$, 2π should be added to ϕ_{high} as there is a transition from $\phi = 2\pi$ to $\phi = 0$ that is invisible due to the partition wall. For the two-chamber experiment, CF derived from the two-chamber experiment is $\mathrm{CF} = 0.50\pi\ \mathrm{g\,mg^{-1}\,mm}$.

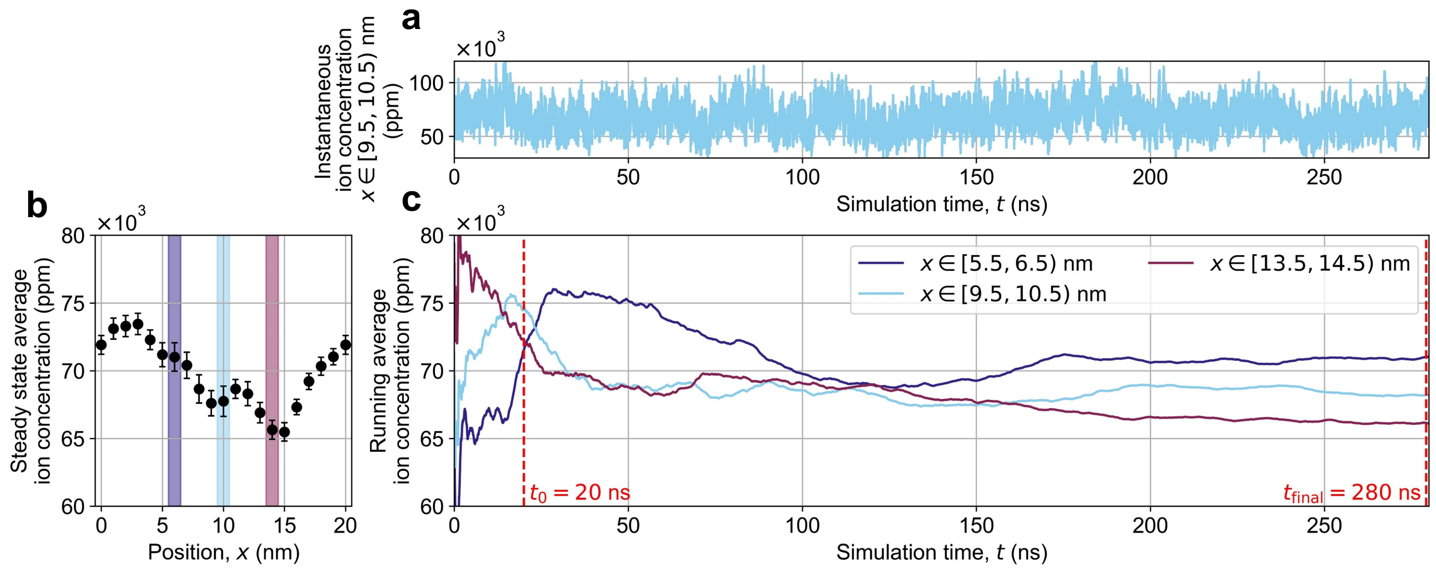

Supplementary Figure 9 | MD simulation: convergence of NaCl brine concentration profile over the time length of the simulation. The ion concentration profile of NaCl brine converges to a measurable steady state condition. The time-averaged ion concentration between simulation time 20 ns and 280 ns is the concentration reported in the main manuscript. The time taken to reach quasi-steady state t_0 is estimated to be 20 ns by the scaling law [4]. For the each highlighted simulation sub-volume ($x \in [5.5, 6.5)$ nm (purple), $x \in [9.5, 10.5)$ nm (light blue), and $x \in [13.5, 14.5)$ nm (mauve), the cumulative running average ion concentration shows reduced fluctuation by $t_0 = 20$ ns and converges over the simulation time interval $[t_0, t_{\text{final}}]$ to the steady state average ion concentration, meaning that the quasi-steady state system has been sampled for a sufficient number of times. This figure shows data for one replicate of a NaCl brine MD simulation, the other two replicates behaved similarly. **a,** The instantaneous local ion concentration in the simulation sub-volume $x \in [9.5, 10.5)$ nm (purple band) is plotted at each time step in the simulation. The instantaneous local concentration has large fluctuations over the full simulation time length, highlighting the intrinsic randomness in molecular scale systems. **b,** The steady state average ion concentration profile at t_{final} over the simulation x-axis is shown with black circle markers. The errors are standard deviations when averaging over the simulation time ([20, 280) ns. Purple, light blue, and mauve shaded regions indicate sub-volumes of the simulation for which a cumulative running average of the ion concentration has been calculated. **c,** Purple, light blue and mauve solid lines show the cumulative running average of the ion concentration of the correspondingly coloured simulation sub-volume. Vertical dashed red lines denote t_0, i.e. the time attributed to reach quasi-steady state, and t_{final}, the total simulation time.

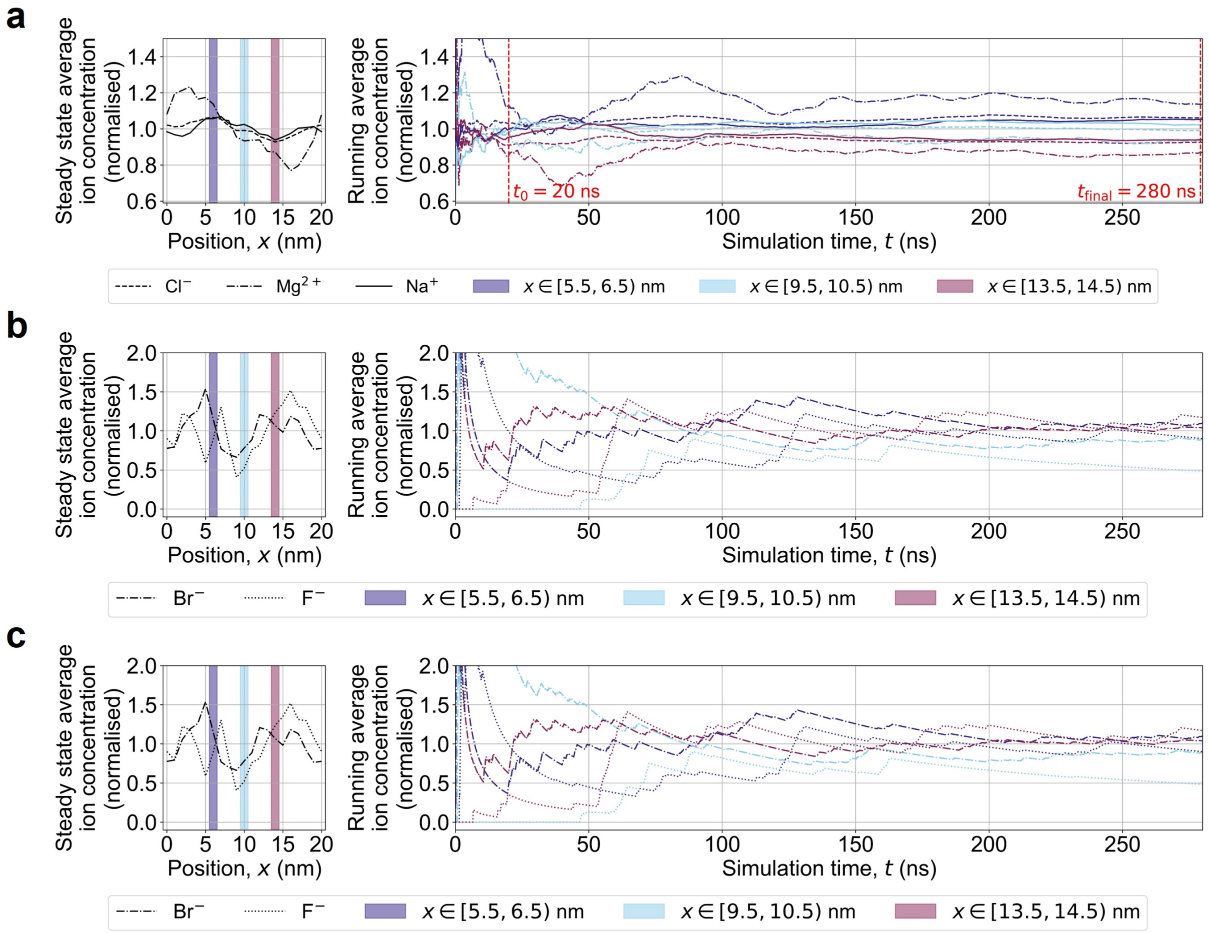

Supplementary Figure 10 | MD simulation: convergence of seawater brine concentration profile over the time length of the simulation. The time-averaged concentration between simulation time 20 ns and 280 ns is the concentration reported in the main manuscript. The individual ion components of the seawater brine are considered separately. Note that this figure shows data for one replicate of a seawater brine MD simulation, the other three replicates behaved similarly. Within each sub-figure, the left plot shows the steady state average ion concentration of the given ions (black lines), and three highlighted simulation sub-volumes ($x \in [5.5, 6.5)$ nm (purple), $x \in [9.5, 10.5)$ nm (light blue), and $x \in [13.5, 14.5)$ nm (mauve)) for which a cumulative running average of the ion concentration has been calculated. The right plot shows shows the cumulative running average of the ion concentration of the correspondingly coloured simulation sub-volume, for each of the given ions. **a,** The ion concentration profiles of Cl^- (dashed line), Na^+ (solid line), and Mg^{2+} (dashed and dotted line) converge to a measurable steady state condition. Each of these ions had significant initial concentrations in the seawater brine solution and therefore no sampling issues were encountered. For the each highlighted simulation sub-volume, the cumulative running average ion concentration shows reduced fluctuation by $t_0 = 20$ ns and converges over the simulation time interval $[t_0, t_{\text{final}}]$ to the steady state average ion concentration. **b,** The convergence of the ion concentration profiles of Ca^{2+} and K^+ to a measurable steady state was hard to characterise due to the sampling issues of very few (six) ions in the total simulation volume. The cumulative running averages of both ions did not smooth over the simulation time frame. Discrete jumps in the cumulative running averages, even after 150 ns of simulation time, highlight the challenge of measuring steady state properties from few ions. **c,** Similarly, the convergence of the ion concentration profiles of Br^- and F^- to a measurable steady state was hard to characterise due to the sampling issues of very few (three) ions in the total simulation volume. The cumulative running averages of both ions did not smooth over the simulation time frame. Discrete jumps in the cumulative running averages, even after 250 ns of simulation time, highlight the challenge of measuring steady state properties from few ions.

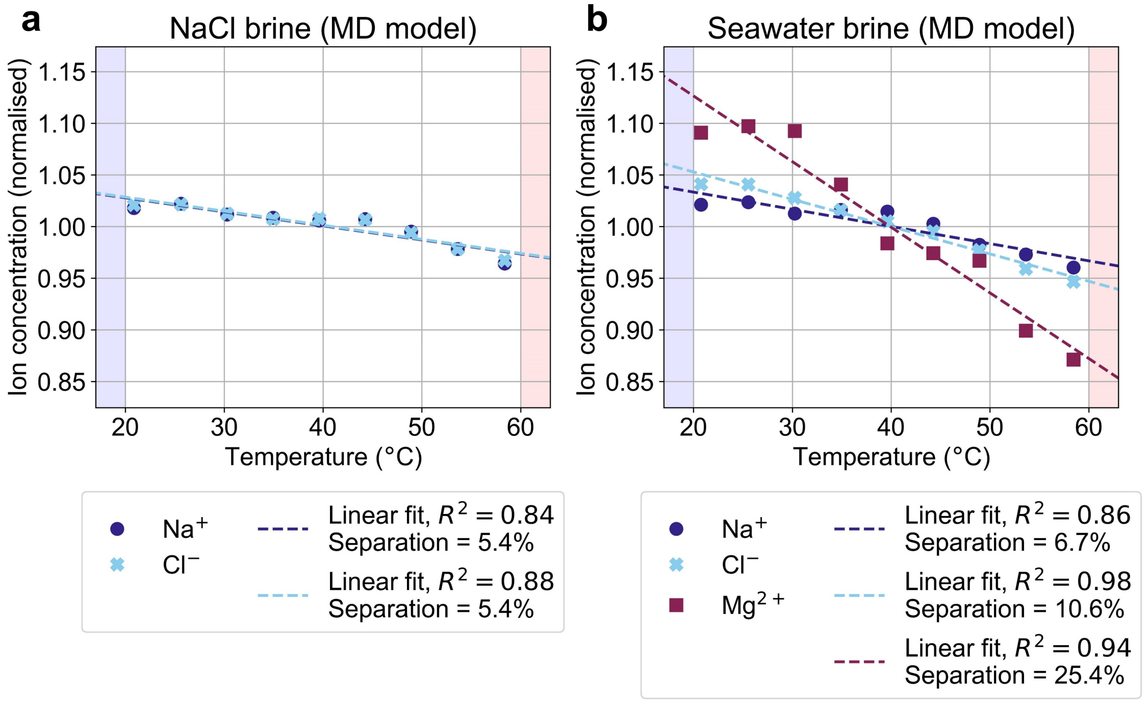

Supplementary Figure 11 | MD simulation: concentration profiles of individual ions in modelled brine of NaCl and seawater. Markers show the concentration over temperature of ions with significant initial concentrations in solution, that is Na^+ (purple circles), Cl^- (light blue crosses), and Mg^{2+} (mauve squares). The temperature range spans 20–60 °C, and is achieved by hot and cold thermostats shown as the shaded red and blue regions, respectively. Ion concentration profiles are normalised to the average concentration of that ion in the solution, to allow comparison of the strength of ion separation. All ions showed thermophobic separation, with concentration increasing as temperature decreases, characterised by linear lines of best fit (dashed lines). An R^2 values is reported for each linear fit in the sub-figure legends. The strength of ion separation is characterised by a separation percentage, that is the difference between the normalised ion concentration at the temperature extremes, $T_{\text{cold}} = 20\,°\text{C}$ and $T_{\text{hot}} = 60\,°\text{C}$, as given by the linear fit, and reported in the sub-figure legends. **a,** Cl^- and Na^+ concentration profiles in modelled NaCl brine. Markers overlap due to electroneutrality. **b,** Cl^-, Na^+, and Mg^{2+} concentration profiles in modelled seawater brine.

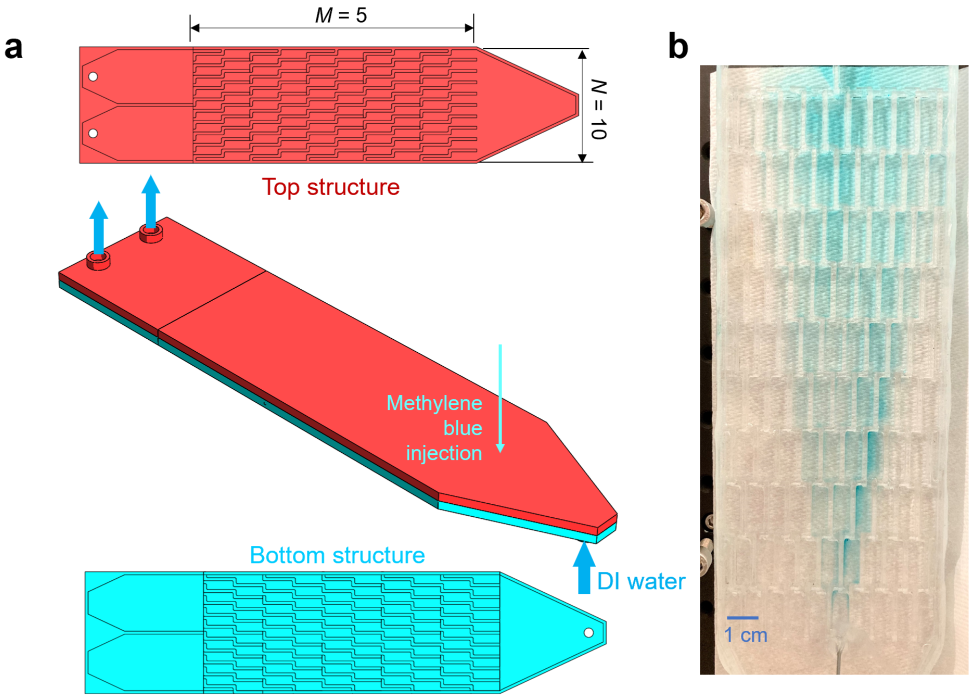

Supplementary Figure 12 | Burgers cascade: a single flow pass multi-channel device. The number of cells in the device is $(M \times N) = (5 \times 10)$. **a,** This Burgers cascade is made from four layers of cast acrylic. The top and bottom layers are walls of 3 mm in thickness while the middle two layers are 1 mm thick. From the bottom up, the first two layers are denoted in blue; the top two layers are denoted in red. The top and bottom walls were laser cut to shape their perimeter and laser-engraved with the shape of the adjacent middle layer. The two middle layers were laser cut and glued to the top and bottom walls separately, forming the blue and red parts. Then the two parts were glued together to form the Burgers cascade. There was one inlet and two outlets for the flow. The methylene blue injection needle was place at inlet of the cell $m = 1, n = 6$. **b,** Photo showing the controlled spread normal to the flow directly of the methylene blue dye. A peristaltic pump was used to slowly pump water into the structure at a flow rate of $10\,\mathrm{mL\,min^{-1}}$ so that the Burgers cascade is entirely filled with liquid without bubbles. The dye was injected with a syringe pump while the water is flowing. Since the density of the dye is larger than water, the spread towards the right exit is more visible than towards the left exit.

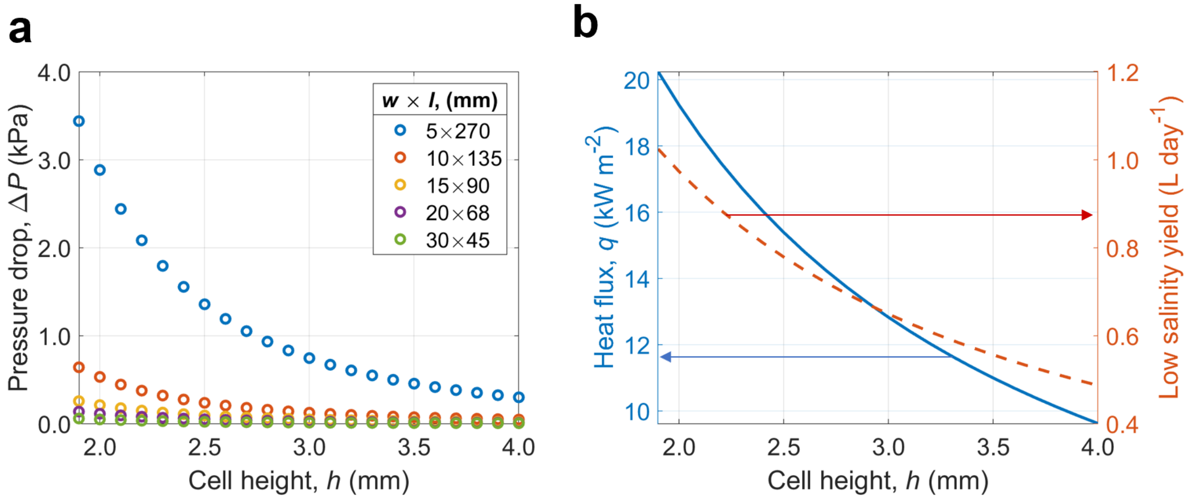

Supplementary Figure 13 | Burgers cascade: pressure drop, yield and heat flux. For a target yield concentration of 5000 ppm with a recovery rate of 10%, the number of cells in the device is $M \times N = 185 \times 20$. A ΔT of 60 K is applied and the Burgers cascade occupies an area of 10 m^2. **a,** The pressure drop varies depending on the individual cell dimensions but does not exceed 4 kPa. **b,** Both the yield of low-salinity water (5000 ppm) and the heat flux decrease with increasing cell height h.

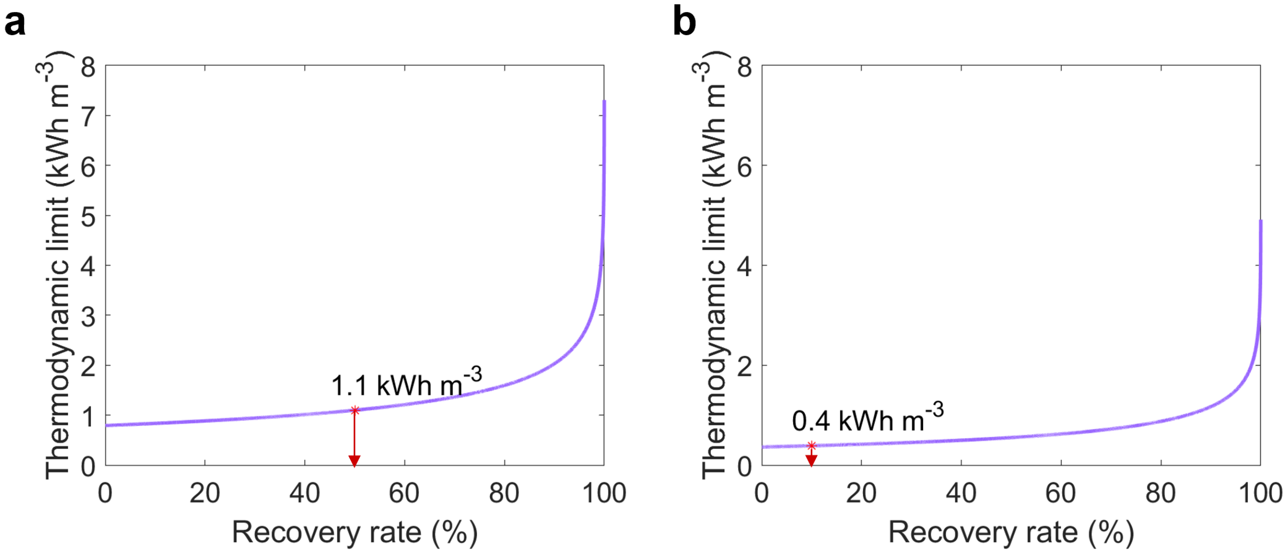

Supplementary Figure 14 | Theoretical minimum energy of separation for desalination. The thermodynamic limit that dictates the theoretical minimum energy of separation is calculated based on the Gibbs free energy, as described in Supplementary Method 8 [5]. **a,** The initial feedwater concentration is 35 000 ppm and the separation is assumed to be complete, i.e. the salt concentration is 0 in the produced fresh water. **b,** The initial feedwater concentration is 30 000 ppm and the yield water concentration is 5000 ppm, which is the same case that is modelled in Supplementary Fig. 13.

Supplementary Table 1 | Ion compositions of seawater brine and NaCl brine solutions modelled in MD simulations. A multi-ion brine solution was constructed to approximate natural seawater, sourced from [6]. To be compatible with the MD simulation system and the TIP3P-FB water model, the multi-ion solution was required to be electrically neutral, contain ions with available parameters that are compatible with the TIP3P-FB water model (restricting the solution to monoatomic ions), and have an integer number of each ion species. The relative concentrations of anion components (Cl^-, F^-, and Br^-) were exaggerated and small cation components (Sr^{2+}) were ignored in the final ion composition to maximise anion diversity and balance the electrical neutrality of the overall solution. Finally, the total concentration of the multi-ion seawater solution was doubled to increase the number of each ion species and therefore improve the sampling. A control NaCl brine solution was constructed with the same molar concentration as the seawater brine.

**Ion species**	**Natural seawater** ($mg\,L^{-1}$)	**Natural seawater** (reduced to volume of simulation) (# ions)	**Ion parameters compatible with TIP3P-FB water**	**Seawater brine used in MD simulations** (# ions)	(Difference from natural seawater)	**NaCl brine used in MD simulations** (# ions)
Cl^-	19000	161	Sengupta et al. [7]	338	110%	325
Na^+	10500	138	Sengupta et al. [7]	260	89%	325
Mg^{2+}	1350	17	Li et al. [8]	33	97%	
$SO_4{}^{2-}$	885	3	Not available			
Ca^{2+}	400	3	Li et al. [8]	6	100%	
K^+	380	3	Sengupta et al. [7]	6	105%	
Br^-	65	0.24	Sengupta et al. [7]	3	1125%	
Sr^{2+}	8	0.03	Li et al. [8]			
$B(OH)_3$	4.6	0.02	Not available			
$Si(OH)_4$	3	0.01	Not available			
F^-	1.3	0.02	Sengupta et al. [7]	3	14460%	
			**Total ion count:**	649		650

Supplementary Table 2 | Linear fitting parameters and Soret coefficients from multi-pass experiments. Multi-pass TDD was performed for both NaCl/H_2O and multi-ion solutions. Different methods were used to measure the species concentration. Linear regression was performed for concentration in the top stream C_{low} and the number of passes n, which yields C_0 as the interception and C_{drop} as the slope. T_{mean} and ΔT_{meas} were the temperature conditions in the experiments. $\Delta C_{\mathrm{walls}}$ and S_{T} can be derived based on Supplementary Method 4.

**Solution**	**Component**	**Methods**	C_0 **(ppm)**	$C_{\mathbf{drop}}$ **(ppm)**	$\Delta C_{\mathbf{walls}}$ **(ppm)**	$T_{\mathbf{mean}}$ (°C)	$\Delta T_{\mathbf{meas}}$ (K)
NaCl/H_2O	NaCl	PSI	29 974.1 ± 52.8	306.5 ± 21.6	1 656.5 ± 116.8	40.84	35.48
NaCl/H_2O	NaCl	PSI	59 929.23 ± 73.3	571.01 ± 29.9	3 086.81 ± 161.6	43.47	31.29
Multi-ion saline solution	Na^+	ICP-AES	14 200.0 ± 292.2	190.0 ± 119.3	1 027.0 ± 664.9	41.49	34.73
		ICP-AES	13 329.4 ± 203.9	111.1 ± 83.2	600.4 ± 449.7		
	Mg^{2+}	ICP-AES	13.4 ± 0.4	0.1 ± 0.2	0.5 ± 0.8		
		ICP-MS	12.1 ± 0.2	0.1 ± 0.1	0.40 ± 0.4		
	Ca^{2+}	ICP-AES	27.6 ± 0.5	0.30 ± 0.2	1.62 ± 1.0		
		ICP-MS	27.5 ± 0.2	0.3 ± 0.1	1.77 ± 0.5		
	All cations	ICP-AES	14200.1 ± 292.2	190.40 ± 119.3	1 029.19 ± 644.9		
		ICP-MS	13 329.4 ± 203.9	111.1 ± 0.583.3	600.4 ± 450.27		

**Solution**	**Component**	**Methods**	$S_{\mathbf{T}}$ (10^{-3} K^{-1})	$S_{\mathbf{T,\ high}}$ (10^{-3} K^{-1})	$S_{\mathbf{T,\ low}}$ (10^{-3} K^{-1})
NaCl/H_2O	NaCl	PSI	1.51	1.65	1.46
NaCl/H_2O	NaCl	PSI	1.65	1.73	1.51
Multi-ion saline solution	Na^+	ICP-AES	2	4	1
		ICP-MS	1	2	0
	Mg^{2+}	ICP-AES	1	3	-
		ICP-MS	1	2	-
	Ca^{2+}	ICP-AES	2	3	1
		ICP-MS	2	2	1
	All cations	ICP-AES	2	4	1
		ICP-MS	1	2	0

Supplementary Table 3 | Comparison of thermodiffusive desalination with other emerging desalination technologies. The desalination performance reported in representative literature for promising emerging technologies is listed. Shaded cells are values calculated from the literature, not directly cited. Nomenclature: initial concentration, C_0; yield concentration,C_{yield}; recovery rate, R_w; thermodiffusive desalination, TDD; metal–organic framework, MOF.

**Ref.**	**Technology**	**Remarks**	C_0 **(ppm)**	C_{yield} **(ppm)**	**Achieved yield**	**Potential yield per day**	**Salt removal per day**	R_w **(%)**	**Similar refs.**
This work	TDD, single-pass channel	A membrane-free, single-phase, thermal desalination process	30 000	29 550	2.88 L day^{-1}	288 L m^{-2} ($M = 1,\ N = 1$)	130 g m^{-2}	50%	–
	TDD, Burgers cascade		30 000	25 000	–	7.9 L m^{-2} ($M = 12,\ N = 6$)	39.5 g m^{-2}	50%	
	TDD, Burgers cascade		60 000	30 000	–	0.95 L m^{-2} ($M = 100,\ N = 15$)	28.5 g m^{-2}	50%	
	TDD, Burgers cascade		30 000	15 000	–	1.00 L m^{-2} ($M = 95,\ N = 15$)	15.0 g m^{-2}	50%	
	TDD, Burgers cascade		30 000	5000	–	0.24 L m^{-2} ($M = 400,\ N = 25$)	5.9 g m^{-2}	50%	
Ou et al. [9] (2020)	Ion adsorption	Adsorbent is a photo-responsive MOF-polymer	35 000<br>10 000<br>1000	$\leq$ 600	1 mL g$^{-1\dagger}$<br>5.6 mL g$^{-1\dagger}$<br>81 mL g$^{-1\dagger}$	139.5 L kg^{-1} (from 2233 ppm to 1179 ppm)	147 g kg^{-1} or 71.67 g m^{-3}	40%<br>79%<br>98%	[10, 11]
Porada et al. [12] (2012)	Wire-based capacitive deionisation	Remove ions by alternately dipping an array of electrode pairs in fresh water and in brine, with and without an applied cell voltage, respectively	1168	$\leq$584.4	50 mL	1.1 L	0.64 g day^{-1}	50%	[13–15]
Kim et al. [16] (2010)	Ion concentration polarisation	Microfluidics High energy efficiency at 3.5 kWh m^{-3}	30 000	$\leq$180	14.4 mL day^{-1}	7100 L m$^{-2\ddagger}$	200 kg m^{-2} day	50%	
Gong et al. [17] (2021)	Solar-driven interfacial evaporation	Energy efficiency is 67.4% (solar energy to latent energy of evaporation)	32 500	$\leq$50	0.3 mL h^{-1} for a 3.1 cm^2 illuminated area	7.9 L m^{-2}, calculated from 0.69 L m^{-2} h^{-1} assuming 8 hours of sunlight	250 g m^{-2}	81%	[18–20]
Ni et al. [21] (2018)		Energy efficiency is 22% (solar energy to latent energy of evaporation)	30 000	$\leq$50	0.39 L day^{-1} for a 0.3 m^2 prototype	2.5 L m^{-2}	75 g m^{-2}	–	
Mao et al. [22] (2020)	Membrane distillation	Feedwater at 60 °C had highest yield	35 000	$\leq$50	1.36 g h^{-1} for the 11.9 cm^2 membrane	326 L m^{-2} assuming 24 hours operation	11.4 kg m^{-2}	–	[23–25]

† The amount of fresh water needed for regeneration of PSP-MIL-53 was determined to be 1.5 mL g^{-1}. It is excluded from the yield.
‡ The scalability is calculated but not demonstrated in the paper.

Supplementary Method 1: Modelling of continuum thermodiffusion

Discretisation of governing equation

The model of thermodiffusive separation in the non-isothermal channel flow was conducted assuming a continuum model [26] (in contrast to the discrete molecular dynamics model described in Supplementary Method 6). The continuum thermodiffusion model is based on the conservation of chemical species (NaCl) in a fluid flow with constant velocity field $\mathbf{u}$. The governing equation is:

$$\frac{\partial}{\partial t}(\rho C) + \nabla \rho \mathbf{u} C = -\nabla \mathbf{J}, \tag{1}$$

where ρ is the local density of the solution ($\mathrm{kg\,m^{-3}}$) and C is the local concentration of the species (in weight fraction or wt%); $\mathbf{u}$ is the velocity field and $\mathbf{J}$ is the mass flux. In the presence of both concentration and temperature gradients, the mass flux $\mathbf{J}$ in a binary solution is [27]:

$$\mathbf{J} = -\rho D \nabla C - \rho C(1-C) D_{\mathrm{T}} \nabla T. \tag{2}$$

The first term on the right-hand side (RHS) describes the mass flux due to isothermal mass diffusion (i.e. Fickian diffusion), which occurs spontaneously in the presence of a concentration gradient ∇C. D is the mass diffusion coefficient, which is always positive because molecules diffuse spontaneously from high to low concentration regions. The second term on the RHS describes the thermodiffusive mass flux, which is likely to occur in the presence of a temperature gradient ∇T. D_{T} is the thermodiffusion coefficient, which may be either positive for thermophobic or negative for thermophilic species. The Soret coefficient S_{T} is defined as $S_{\mathrm{T}} \equiv D_{\mathrm{T}}/D$. The same as with D_{T}, S_{T} could be either positive or negative, which corresponds to thermophobic or thermophilic species, respectively (because D is always positive).

The fluid is a NaCl/H_2O solution, with D and S_{T} being experimental values reported in the literature [27]. Under the assumption of steady-state thermodiffusion, for a Soret cell without advection [1] and assuming no lateral boundary effects as in Supplementary Fig. 2a, Eq. (1) is reduced to: $0 = \rho D \nabla C_y - \rho C(1-C) D_{\mathrm{T}} \nabla T_y$. When considering transient thermodiffusion within a Soret cell, we apply the mass conservation of the chemical species as in Eq. (1) in the form:

$$\rho \frac{\partial C}{\partial t} = \frac{\partial}{\partial y}\left[-\rho D \nabla C_y - \rho C(1-C) D_{\mathrm{T}} \nabla T_y\right]. \tag{3}$$

Next, vertical thermodiffusive separation in a horizontal Poiseuille flow with a binary solution was modelled. The model is shown in Supplementary Fig. 2b. The fluid flows in the x-direction between two parallel plates held isothermal at different temperatures. Our desalination method splits the channel flow into two streams of equal flow rate at the channel outlet. Note that the velocity is not modelled but set as an input in our modelling based on the analytical solution of a fully-developed plane Poiseuille flow. We then may apply multiple channels to scale up the separation process. Here, we assume advection in the x direction with a fully developed velocity profile. In the y-direction, governing equation remains the same as Eq. (3), i.e. unaffected by the advective term, while in the x-direction

mass conservation is given by

$$\rho \frac{\partial C}{\partial t} + \frac{\partial}{\partial x}(\rho u C) = \frac{\partial}{\partial x}(-\rho D \nabla C_x). \tag{4}$$

A fully-implicit finite volume method (FVM) was employed to solve the governing equations, as described in [28]. The discretisation equation is given below in Eq. (6) [28]. For calculation of concentration at the grid interface, we consider the Péclet number, Pe, which is defined as the advective transport rate to diffusive transport rate:

$$\mathrm{Pe} \equiv \frac{uL}{D/L^2}, \tag{5}$$

where L is the characteristic length. In the x direction, $\mathrm{Pe}_x = u(x)L_{\mathrm{cha}}/\left(D/L_{\mathrm{cha}}^2\right) \gg 1$, thus an upwind scheme is used. While in the y direction, $\mathrm{Pe}_y = u(y)h/\left(D/h^2\right) = 0$, so a piecewise-linear profile is used. The coefficients at the control volume faces are calculated as the harmonic mean of adjacent grid values. Grid and time-step dependence studies were performed. Grid size between 10 µm and 100 µm was tested and 20 µm was deemed to be precise enough. A time step independence study was conducted varying the time step between 0.01 s and 1 s; a time step of 0.1 s was deemed appropriate.

$$a_P C_P = a_E C_E + a_W C_W + a_N C_N + a_S C_S + d, \tag{6}$$

where

$$\begin{aligned}
a_E &= \frac{D_e}{\Delta x} \\
a_W &= \frac{D_w}{\Delta x + u_w} \\
a_N &= D_{\mathrm{T},n} \nabla T_y \left(\frac{1}{2} - \frac{C_N}{4}\right) + \frac{D_n}{\Delta y} \\
a_s &= -D_{\mathrm{T},s} \nabla T_y \left(\frac{1}{2} - \frac{C_S}{4}\right) + \frac{D_s}{\Delta y} \\
d &= \frac{\Delta x \Delta y}{\Delta t} C_P^0 \\
a_P &= \frac{\Delta x \Delta y}{\Delta t} + u_e + \frac{D_e}{\Delta x} + \frac{D_w}{\Delta x} + \frac{D_n}{\Delta y} + \frac{D_s}{\Delta y} \\
&\quad - D_{\mathrm{T},n} \nabla T_y \left(\frac{1}{2} - \frac{C_P}{4} - \frac{C_N}{2}\right) + D_{\mathrm{T},s} \nabla T_y \left(\frac{1}{2} - \frac{C_P}{4} - \frac{C_S}{2}\right)
\end{aligned}$$

Verification and validation

Our diffusion-only modelling results (without advection) were first compared against the corresponding analytical solution:

$$C(y) = \left[1 + \exp\left(\frac{y S_{\mathrm{T}} \Delta T}{h} + \epsilon\right)\right]^{-1}, \tag{7}$$

where ϵ is a constant that can be determined by mass conservation. The analytical solution can only be derived when the thermophysical properties are constants and the temperature gradient is constant, thus it is a relatively accurate representation of the physical model only when the temperature difference between top and bottom is small. The analytical solution for $T_{\text{mean}} = 40\,^{\circ}\text{C}$ and $\Delta T = 40\,\text{K}$ is $C(y) = [1 + \exp(84.4y + 3.46)]^{-1}$.

The comparison between the analytical and modelling result at steady state in the absence of advection is shown in Supplementary Fig. 3. Since the analytical solution cannot account for the temperature dependence of the coefficients, in the simulation results of **a** the coefficients were taken as constant at the mean temperature. A good agreement was obtained between the analytical and numerical solutions. The simulation result with experimentally measured temperature dependence of S_{T} and D [27] is shown in **b**. Due to the temperature dependence, the concentration profile is non-linear. The concentration gradient is larger in the upper half of the channel where the temperature is higher, and S_{T} is also larger.

To validate the numerical model capturing time dependent behaviour, the one-dimensional transient FVM results for different binary solutions were compared against experimental results reported in the literature [1, 2] using the reported thermodiffusion coefficients and experimental conditions (including Soret cell height h, initial concentration C_0, ΔT and T_{mean}) provided in those papers. A good agreement between the simulated concentration profile (and concentration difference) and the experimental results, especially at steady state, was obtained, as shown in Supplementary Fig. 4. This brings confidence to our simulation method. In Supplementary Fig. 4a, for the isothermal diffusion phase, the isothermal condition is not applied instantaneously in the experiment. In addition, the abrupt change in temperature within the Soret cell could have produced vortices, especially close to the sidewalls where temperature gradients are not horizontal (non-adiabatic condition). These effects could have contributed to the discrepancy between experimental and simulation results based on ideal boundary and initial conditions. In Supplementary Fig. 4b, the discrepancy for transient concentration profiles could be explained by the scattered red dots that fall below the general trendline in Supplementary Fig. 4a, i.e. the transient experimental concentration profiles may be inaccurate. For Supplementary Fig. 4c, simulation results agree reasonably well within the experimental data, especially in the central part of the Soret cell away from the edges.

Discussion

The validated model can then be used to predict thermodiffusive separation in a Soret cell without advection. We define the time required to reach 95% of a complete thermodiffusive separation between channel walls as the "thermodiffusion separation time" τ_{th}, i.e. $\Delta C_{\text{walls}} = 0.95\,\Delta C_{\text{steady}}$ where ΔC_{steady} is the maximum possible separation achieved when $t \to \infty$ in a convectionless cell. Simulation results in Supplementary Fig. 5a confirm that for aqueous electrolytes such as seawater, the thermodiffusion time constant τ_{th} can be accurately calculated as

$$\tau_{\text{th}} = \frac{h^2}{\pi^2 D}, \tag{8}$$

which has been experimentally observed [1, 29]. Here, h is the channel height and D the mass diffusion coefficient. Equation (8) does not depend on the thermodiffusion phenomenon itself when $S_\mathrm{T} \ll 0.1\,\mathrm{K}^{-1}$. Supplementary Fig. 5b shows the transient thermodiffusive separation without advection in terms of the concentration difference between walls (ΔC_walls) as a function of time. The separation rate is first large but then plateaus, converging to ΔC_steady. Furthermore, τ_th is generally independent of ΔT. Next, we model thermodiffusive separation in a channel with advection. Supplementary Fig. 5c shows the fully-developed steady-state concentration profile ΔC_dev for a range of different temperature differences across a 1 mm high channel for the planar Poiseuille flow (as in the TDU experiments). Interestingly, a high degree of similarity exists between the concentration profile that is far into the channel (e.g. 500 mm) and the case without advection, i.e. $\Delta C_\mathrm{dev} \approx \Delta C_\mathrm{steady}$. ΔT is a major factor that determines the degree of thermodiffusive separation. It is noted that for the condition of $\Delta T = 60\,\mathrm{K}$, the ΔC_walls is 11% of C_0, but after accounting for the effect of the parabolic velocity profile (with no-slip boundary condition), the concentration difference between the top half and the bottom half of the saline steams is 4.7% of C_0. As S_T is generally temperature dependent with the existence of an inversion temperature T_0 where $S_\mathrm{T}(T_0) = 0$, the choice of the temperature range is important. Though larger ΔT generally results in larger ΔC_walls, getting close to or even encompassing T_0 within the applied temperature range may result in marginal improvement in separation. The Schmidt number (defined as $\mathrm{Sc} \equiv \nu/D$, where ν is the kinematic viscosity) for aqueous NaCl is ca. 599 at 25 °C, i.e. the mass transfer is advection dominant in the x direction. Therefore, due to a Poiseuille flow in the x direction having a high value of Sc (and the flow being laminar), one-dimensional modelling of mass diffusion in the y direction is a good approximation for evaluating ΔC_walls and τ_th.

Supplementary Method 2: Design rationale for a thermodiffusive desalination channel

Based on the knowledge gained on thermodiffusive separation in a laminar channel flow from continuum modelling (Supplementary Method 1), we can design a thermodiffusive desalination channel. First, dimensionless numbers and entrance lengths were calculated to confirm a laminar flow regime, a fully-developed velocity profile, and quasi-linear temperature profiles. Next, the effect of the TDU channel dimensions on the flow rate (yield) and the heat flux (energy consumption) were analysed. It was found that the thermal energy consumption per unit of desalinated water produced is independent of the channel dimensions. Finally, considering practical limits such as heating and cooling power capacity (e.g. using water baths as in our experiment) and practical device dimensions, the TDU channel size was determined.

Through our CFD modelling detailed in Supplementary Method 1, we calculate the thermodiffusion time constant τ_th from Eq. (8), and the results are shown in Supplementary Fig. 5a. We noticed that τ_th is relatively large even for a small h, as shown in Supplementary Fig. 5b, in a 1 mm height cell, τ_th is around 90 s. This imposes a limit on the flow speed u_dev along the channel if we are aiming for a fully-developed concentration profile. The fully developed mean velocity in the channel then becomes

(substituting Eq. 8)

$$u_{\text{dev}} = \frac{L}{\tau_{\text{th}}} = \frac{L\pi^2 D}{h^2}. \tag{9}$$

Another important aspect to consider when designing a parallel-plate thermodiffusive separation channel is that the established concentration profile in the y-direction due to thermodiffusion remains undisturbed, i.e. the fluid flow is laminar and without buoyancy. Since there exists a positive temperature gradient across the channel height, a negative Rayleigh number (Ra) is produced yielding a stabilising condition for positive values of S_{T} [1]. The fluid motion is created only by forced convection along the TDU channel with the pump controlling its flow rate. The Reynolds number (Re) is calculated from its definition $\text{Re} \equiv \rho u L_{\text{c}}/\mu$, where the characteristic length L_{c} is the channel height h for a parallel-plate channel and when substituting the flow speed calculated in Eq. (9), the Reynolds number for a fully-developed flow becomes

$$\text{Re}_{\text{dev}} = \frac{\pi^2 \rho L D}{h\mu}. \tag{10}$$

Thus, Re_{dev} can be plotted for different channel dimensions as shown in Supplementary Fig. 6a. For a range of channel dimensions, we see that Re_{dev} is well below the critical Reynolds number threshold, thus confirming that a laminar flow regime is caused when imposing the constraint of τ_{th}, Eq. (8).

Furthermore, the entrance length can be solved analytically since Re_{dev} is known. The normalised hydrodynamic entrance length is $L_{\text{v}}/L = 0.04\text{Re}_{\text{dev}}h/L$ [30]. Substituting Eq. (10), it becomes

$$\frac{L_{\text{v}}}{L} = \frac{0.04\rho\pi^2 D}{\mu}, \tag{11}$$

which is less than 0.1% for the corresponding thermophysical properties of water in the operating temperature range. Furthermore, from the definition of Prandtl number Pr, the normalised thermal entrance length is $L_{\text{T}}/L = \text{Pr}L_{\text{v}}/L$. Since $\text{Pr} < 15$ for liquid water under normal pressure, L_{T}/L is always smaller than 1%. From the analytical calculation, we see that the normalised entrance lengths for momentum L_{v}/L and heat L_{T}/L are both independent of channel dimensions and are less than 1% of the total channel length. Thus, both a fully-developed plane Poiseuille flow through most of the channel and a quasi-linear temperature profile along the channel height are valid assumptions.

We then consider two important design parameters for the separation device: the areal flow rate Q'_{dev}, which is the volumetric flow rate per unit channel width ($Q'_{\text{dev}} = Q/w$), and the areal heat flux q'_{dev}, which is the heat transfer rate per unit channel width ($q'_{\text{dev}} = q/w$). The areal flow rate is set such that a near full separation occurs at the channel outlet, i.e. $Q'_{\text{dev}} = h \times u_{\text{dev}} = hL/\tau_{\text{th}}$ (recall Eq. 9). Combining with Eq. (8), the areal flow rate becomes

$$Q'_{\text{dev}} = \frac{\pi^2 D L}{h}. \tag{12}$$

Moreover, from Fourier's first law of conduction, i.e. the conductive heat flux being $q'' = -k\,\mathrm{d}T/\mathrm{d}y = q/(Lw)$, the areal heat flux normal to the hot and cold channel walls becomes

$$q'_{\text{dev}} = -k\frac{\Delta T L}{h}, \tag{13}$$

where k is the thermal conductivity of the saline solution. Hence, under the conditions of fully-developed concentration profile and from Eqs. (12) and (13), the thermal energy consumption per unit of desalinated water is

$$\dot{E}_{\text{th}} = \frac{q'_{\text{dev}}}{Q'_{\text{dev}}} = -k\frac{\Delta T}{\pi^2 D}, \tag{14}$$

which is independent of the channel dimensions. The preferred design features of a TDU are large volumetric flow rate Q with a large species separation ΔC. Based on the above discussions, some relationships are now clear. For the volumetric flow rate Q through a channel of width w, the following expression is obtained

$$Q = Q'_{\text{dev}} w \propto \frac{Lw}{h}, \tag{15}$$

where Eq. (12) was substituted. For the concentration difference between walls, the following expression can be obtained from Supplementary Fig. 5b:

$$\Delta C_{\text{dev}} \propto \Delta T. \tag{16}$$

In addition, another limiting factor is the thermal power supply from the water bath in the lab, P_{wb}, or from any other heat source (such as industrial waste heat or heat from the surrounding environment). The heat transfer rate q normal to the channel upper and lower walls becomes:

$$q = q'_{\text{dev}} \times w = k\frac{\Delta T L w}{h} \propto \frac{\Delta T L w}{h}, \tag{17}$$

which is obtained directly from Eq. (13) and the definition of areal heat flux. Comparing Eq. (17) with Eqs. (15) and (16), we arrive at the conclusion that the overall salt removal rate of $Q\Delta C_{\text{dev}}$ is proportional to the heat flux through the channel and hence constraint by the power input P_{wb}.

For the purpose of reducing the error in concentration measurement, we aim for large ΔC without an excessively small Q. Thus, a rather large ΔT of 60 K is chosen. Based on the power rating of the water bath (ca. 415 W) and the thermal conductivity of saline water, the channel dimensions must satisfy the following condition

$$\frac{Lw}{h} \leq \frac{P_{\text{wb}}}{\Delta T k} = 11.5\,\text{m} \tag{18}$$

To minimise the risk of burning hazards, instead of setting the lower wall temperature to 20 °C (hot wall to 80 °C), we set the cooling temperature to 10 deg °C (hot side at 70 °C), which yielded the cooling capacity of 415 W. This configuration slightly reduces the separation due to the lower mean temperature (Fig. 2a). One important condition and assumption for all the above numerical and analytical work is the existence of a fully-developed plane Poiseuille flow, i.e. $u_x = u(y)$ and $u_y = u_z = 0$. To keep this condition, we defined an aspect ratio $r = w/h = 20$. In this case, in the z direction, the boundary layer thickness is approximately the same as h [31], which is only around 5% of w and the assumption $u = u(y)$ is still acceptable. Thus, the length of the TDU is calculated as $L = \frac{P_{\text{wb}}}{\Delta T k} \times \frac{1}{r} \approx 0.5\,\text{m}$ (substituting Eq. 18). The flow rate is calculated as $12.4\,\text{mL}\,\text{min}^{-1}$.

The above analysis was limited to the scenario where the concentration profile is nearly fully developed in the TDU channel. Next, we explore the possibility of having an incomplete separation within the

TDU which may increase the yield (albeit with a lower separation, which may be further processed with the TDU circulation strategy in Fig. 3). In Supplementary Fig. 6b, we define the separation ratio as the ratio of concentration difference at the channel outlet ΔC_{out} to that of a fully developed flow ΔC_{dev}, i.e. $\Delta C_{\text{out}}/\Delta C_{\text{dev}}$. The separation ratio is plotted as a function of the areal flow rate Q'. It is shown that for a constant channel height h, the areal flow rate can be doubled if we aim only for a separation ratio of 0.8 instead of 1. The drop in separation can be justified if increasing the yield is more desirable (e.g. in irrigation applications with crops having a high salinity tolerance). Until this point, the TDU dimension and operation conditions have been designed: $h = 1\,\text{mm}$, $L = 0.5\,\text{m}$, $w = 20\,\text{mm}$ with $\Delta T = 60\,\text{K}$ and $Q \approx 10$ to $16\,\text{mL}\,\text{min}^{-1}$. Based on the above parameters, we confirm that $\text{Re} < 10$ and both L_{v} and L_{T} remain negligible relative to the total channel height L_{cha}. In addition, in the x direction, Pe_x is in the magnitude of 10^3, which means the effect of Taylor dispersion is pronounced and the effective mass diffusion along the channel is enhanced by the shear. The high degree of agreement between experiments and simulations (Figs. 2a,b and 3a,b), which utilise an upwind scheme in the x direction and piecewise scheme in the y direction, also support this assessment.

Supplementary Method 3: Measurement of thermodiffusive separation

3.1 Measurement methods

In Method A (Supplementary Fig. 8b), we used the same method as Torres et al. [3] when measuring Fickian diffusion coefficient D by observing the concentration field between a high- and a low-concentration solution. Essentially, in Supplementary Fig. 8b, when assuming a linear relationship between the unwrapped phase difference ψ and the concentration difference ΔC, the contrast factor $\text{CF} = \frac{\partial \psi}{\text{OP}\partial C}$ can be calculated as $\frac{\Delta \psi}{\text{OP}\Delta C}$, where OP is the optical path of the test cell. If CF is first determined with solutions of known ΔC, then the concentration difference between two unknown solutions can be calculated from ψ extracted from the unwrapped PSI images.

In Method B (Supplementary Fig. 8c), we eliminate the mixing that occurred during the injection process by placing the solutions separately in a two-chamber cell as shown in Supplementary Fig. 8c. With the two solutions separated, the interference data previously observed (i.e. discontinuities in the phase-shifted data shown in Supplementary Fig. 8b) disappear. In this technique, due to the partitioning wall between the two chambers, the numbers of periods between the two solutions cannot be directly determined visually. Thus, it is important to reduce the OP until there are no discontinuous changes of ϕ between the two solutions with its value less than one period (i.e. $\psi_{\text{high}} - \psi_{\text{low}} = \phi_{\text{high}} - \phi_{\text{low}} < 2\pi$). Since CF is positive, $\Delta\psi = \psi_{\text{high}} - \psi_{\text{low}}$ should always be positive. If $\phi_{\text{high}} - \phi_{\text{low}} < 0$, then they are not in the same period and 2π should be added when calculating $\Delta\psi$. The calculation of the phase difference using Method B should follow Eq. (19). We noticed that the CF measured using the two-chamber methods is slighter higher, indicating that the mixing during injection (method shown in Supplementary Fig. 8b) introduced some inaccuracies. All binary NaCl/H_2O solution concentration differences in the paper were measured with Method B, and Method A was used to ensure the $\psi_{\text{high}} - \psi_{\text{low}} < 2\pi$.

$$\Delta\psi = \begin{cases} \phi_{\text{high}} - \phi_{\text{low}}, & \text{if } (\phi_{\text{high}} - \phi_{\text{low}}) > 0 \\ \phi_{\text{high}} - \phi_{\text{low}} + 2\pi, & \text{if } (\phi_{\text{high}} - \phi_{\text{low}}) < 0 \end{cases} \tag{19}$$

3.2 Measurement accuracy

To test the accuracy of our device, we prepared six sets of standard solutions of NaCl/H_2O with known concentration differences. The six sets of solutions prepared had a mean concentration of $C_{\text{mean}} = 25\,000$, 30 000, and 35 000 ppm with $\Delta C/C_{\text{mean}} = 0.02$ and 0.03. Each standard solution was prepared gravimetrically with a total solution mass of 50 g ± 1 mg and a salt mass of around 1.5 g ± 1 mg. Based on ΔC calculated from the sample preparation process and $\Delta\psi$ calculated using Eq. (19), CF was calculated as 1.66 rad g $\text{mg}^{-1}\,\text{mm}^{-1}$ with an error δCF of 0.05 rad g $\text{mg}^{-1}\,\text{mm}^{-1}$.

Thus, we use PSI to measure the concentration difference with the relationship:

$$\Delta C = \frac{\Delta\psi}{\text{OP}\,\text{CF}}. \tag{20}$$

OP is a fixed value as the equipment is unchanged throughout the process. The relative error $\delta\Delta C/\Delta C$ is calculated through error propagation:

$$\frac{\delta\Delta C}{\Delta C} = \left[\left(\frac{\delta\Delta\psi}{\Delta\psi}\right)^2 + \left(\frac{\delta\text{CF}}{\text{CF}}\right)^2\right]^{0.5} \tag{21}$$

The error in the unwrapped phase difference $\delta\Delta\psi$ is the standard deviation when $\Delta\psi$ is obtained for each pair of pixels using Eq. (19) for each individual measurement. The relative error in the contrast factor $\delta\text{CF}/\text{CF}$ was calculated from six sets of standard samples and is 0.030. Thus, for a typical $\frac{\delta\Delta\psi}{\Delta\psi}$ value that is around 10%, the propagated error for the $\frac{\delta\Delta C}{\Delta C}$ is around 10.5%. This corresponds to the vertical error bars in Figs. 2a,b and 3a,b.

Supplementary Method 4: Temperature profile in separation channel

Despite aiming for a constant temperature difference of 60 K in the design process, we noticed a large variation of the temperature profile along the channel. Thus, we experimented with three different water circulation profiles, one parallel flow configuration and two counter flow configurations, each with the same ΔT_{set}. Here, ΔT_{set} is the temperature difference between the set temperatures in the water baths, while the measured temperature difference ΔT_{meas} is lower than that value. For $\Delta T_{\mathrm{set}} = 60\,\mathrm{K}$, values of ΔT_{meas} varied between 31 to 37 K. For $\Delta T_{\mathrm{set}} > 60\,\mathrm{K}$, the heat exchange between the two water baths seemed too large, resulting in a marginal increase in ΔT_{meas}. The $\Delta C_{\mathrm{walls}}$ is shown in Supplementary Fig. 7. Comparing the two counter flow configurations, we see that the water flow has little impact on the temperature profile, i.e. the Counter Flow 2 temperature profile is almost the mirrored version of Counter Flow 1. However, when hot water is circulating in the opposite direction to the saline water in the channel (Supplementary Fig. 7a), the T_{mean} is higher closer to the channel exit, which is equivalent to larger S_{T}. Therefore, a larger ΔC can be obtained.

When comparing the counter flow and the parallel flow configurations, we see that at a very small flow rate of $1\,\mathrm{mL\,min^{-1}}$, the parallel flow configuration can give slightly larger $\Delta C_{\mathrm{walls}}$ at the channel exit. However, when the flow rate is higher, a relatively constant ΔT_{meas} is preferred. We see in Supplementary Fig. 7c that the ΔT_{meas} is increasing from $x = 0.1\,\mathrm{m}$, but a slightly larger flow rate means there is not enough time for the concentration profile development to catch up with the varying temperature profile. Based on these observations, we chose the configuration in Supplementary Fig. 7a for the multiple-pass experiment. The temperature profile along the channel wall can be affected by bubbles accumulating on the heat exchange surface. Due to buoyancy, bubbles appeared more frequently on the top surface of the cold (bottom) water circulation cavity (as indicated in Supplementary Fig. 1b). For nearly identical wall temperature profiles, the thermodiffusive separation was found to have a good repeatability based on four trials where the set $T_{\mathrm{mean}} = 40\,^{\circ}\mathrm{C}$ and $\Delta T = 50\,\mathrm{K}$ yielded $\Delta C = (429 \pm 49)$ ppm. The error is within the accuracy of our in-house PSI concentration measurement system.

Supplementary Method 5: Multi-component saline water re-circulation

Despite the limited data available to determine the thermodiffusive behaviour of a multi-ion electrolyte, we believe it is essential to demonstrate the concept of TDD with seawater. Artificial seawater was produced from sea salt harvested using solar evaporation. The artificial seawater is passed through the TDU multiple times and concentration of different ions are already plotted in the main text. Single-wavelength PSI cannot distinguish between different ions. Thus ICP-AES and ICP-MS were performed by two different labs at ALS and ANU labs, respectively. We noticed that different calibration and measurement procedures can produce different results. In addition, the occasional variations of certain data points made it difficult to extract data from each pair of top and bottom solution from a single pass. Thus it is important to derive a linear fit for $C_{\mathrm{drop,\,exp}}$ as a function of the number of passes. Then a reasonable estimate of the concentration difference between the boundaries can be derived: from C_{drop} and C_0 derived from the linear fits. When assuming constant thermophysical properties, the analytical solution Eq. (7) can be derived. Considering the concentration profile $C(y)$ that is derived from the analytical solution with a parabolic velocity profile and equal flow rate into each stream at the outlets,

the salinity drop C_{drop} is around 18.5% of ΔC between top and bottom boundaries (Fig. 2d). After deriving ΔC from C_{drop}, S_{T} can be calculated.

The mass flux $\mathbf{J}$ of component i in the presence of thermodiffusion for a multi-component solution is:

$$\mathbf{J}_i = -\rho \left[\sum_{k=1}^{n-1} D_{ik} \nabla C_k + D'_{\text{T},i} C_i (1 - C_i) \nabla T \right], \quad i = 1, ..., n-1 \tag{22}$$

where ρ is the density of the solution, D_{ik} denotes the "cross-term" diffusion coefficients as the diffusion of species k can induce the diffusion of species i due to inter-molecular interactions, and $D_{ik} = D_{ii}$ when $k = i$, which describes the dependence of flux of the component i on its own concentration gradient. D_{ii} is referred to as the "main term" diffusion coefficient. In addition, each species has its own thermodiffusion coefficient $D'_{\text{T},i}$. Since data on the cross-terms are not able to be calculated due to the large relative errors in ICP measurements, in an effort to derive $D'_{\text{T},i}$, there are two options.

First, we can treat the multi-component electrolyte as a combination of different binary aqueous solutions. The results for "Na^+", "Mg^{2+}", and "Ca^{2+}" are listed for the multi-ion solution in Supplementary Table 2. The mass flux of ion i, $\mathbf{J}_i$, is independent from the concentration gradient of any other species. That is, there are null cross-term diffusion coefficients $D_{ik} = 0$ when $k \neq i$. Then, $S_{\text{T},i}$ and $D'_{\text{T},i}$ can be written as a function of each other and of D_{ii}, whose values are that of binary solutions reported in the literature. In this study, we found the optimal value of S_{T} in the range of $[0, 1]$ K^{-1} using a single-variable optimisation technique called the golden-section method [32]. The constant ϵ in Eq. (7) can be solved numerically based on mass conservation:

$$\int_0^h C(y) \text{d}y = C_0 h. \tag{23}$$

The second way to interpret multi-component electrolyte diffusion is to make an assumption that ions move collectively as a single species. The results are listed for "All cations" in Supplementary Table 2. In this case, we treat the multi-ion solution as a binary solution of ions and water. S_{T} can also be extracted for this binary solution by applying the golden-section method to obtained experimental data.

We prefer the simplification of treating seawater as a binary mixture of water and ions because we are only interested in the overall removal rate of all the ions instead of individual ion behaviour. Through molecular dynamics simulation, we found that all species in the seawater move collectively, as has been pointed out in the section 'TDD at the molecular level'. We believe the likely cause is the strong electrostatic coupling in ionic electrolyte, which makes it different from the thermodiffusive behaviour of some hydrocarbon mixtures where one component show thermophilicity while the other two show thermophobicity [33]. Therefore, seawater can be effectively treated as binary mixture of ions and water.

Another reason for simplification is that the calculation of these cross-diffusion coefficients rely heavily on the accurate quantification of the concentration of each species in the solution. However, it is not quite possible to perform this task for seawater. For aqueous electrolytes, its composition cannot be viewed as NaCl, $MgSO_4$ etc. but as Na^+, Cl^-, Mg^{2+}, ${SO_4}^{2+}$ because the ionic bounds are broken

and the cations and anions are separately surrounded by water molecules. This means seawater is essentially a twelve-component (or even more) mixture. ICP-OES or ICP-MS can only roughly measure the concentration of cations and we won't have knowledge of the anion concentrations. In addition, the working principle for the ICP methods is that it measures the intensity of the flame at different wavelengths so that the concentration of different cations can be determined. For seawater, Na^+ is overwhelming abundant, making measuring the slight changes in the light intensity at other wavelengths difficult. Despite many communications and iterations with the ICP lab on campus, we could not achieve a better accuracy with the ICP measurements. That means the method of physical extraction then measure the sample concentrations is not possible. The other way is to use non-intrusive optical method. However, two-wavelength system is need for ternary solution [34] and for each component added, a different wavelength has to be added to the optical system. Moreover, there is the exponentially increasing workload that's related to contrast factor measurement [34]. So far, to the best of our knowledge, when it comes to thermodiffusion, the cross-diffusion coefficients are only calculated for ternary solution in published data and there is a group trying to characterise quaternary solutions with a three-wavelength laser. For a twelve-solution mixture, we cannot quite think of possible ways of arranging eleven different lasers on an optical table and we believe at this point of time, it is not possible to extract the cross-diffusion coefficients for each component in seawater.

Supplementary Method 6: Molecular dynamics modelling

A model of thermodiffusive separation of multi-ion solutions in a non-isothermal environment was conducted using a discrete molecular dynamics model. The MD simulations were performed by Nanoscale Molecular Dynamics (NAMD) simulation software [35], following the MD framework for assessing thermodiffusion in aqueous electrolytes published in our previous work [36]. All deviations from the previous methodology are detailed below.

6.1 Model setup

NaCl brine and seawater brine solutions, each consisting of approximately 14 000 water molecules and 650 ions, were contained within a rectangular prism of 20 × 5 × 5 nm with periodic boundary conditions. Structure and geometry parameters for the TIP3P-FB water molecules were sourced from [37]. TIP3P-FB compatible monovalent and divalent ion parameters were sourced from [7] and [8] respectively, having been fit to recreate hydration free energy. These molecule parameters accurately recreate thermodiffusion in aqueous NaCl solutions [36]. A multi-ion brine solution was constructed to approximate natural seawater while remaining compatible with the MD simulation set up. Ion compositions of the seawater brine and comparable control NaCl brine solutions are shown in Supplementary Table 1.

6.2 Temperature regulation

A quasi-linear temperature profile was achieved across the x-axis using volume defined thermostats designed by [38] with no explicit energy conservation algorithm, as described in [36].

6.3 Simulation time step and non-bonded interactions

All parameters were equivalent to those used in [36], except for the grid size of the particle mesh Ewald scheme that was altered to $\{X = 216, Y = 54, Z = 54\}$ to fit the simulation box dimensions.

6.4 Simulation systems and equilibration

Initial configurations of the seawater and NaCl brine solutions had ion molecules placed randomly in the simulation volume using the Packmol software [39] and water molecules placed randomly with the VMD plugin `solvate`. The dilute concentrations of Mg^{2+}, Ca^{2+}, K^{+}, F^{-}, and Br^{-} ions were placed independently to ensure an approximately uniform distribution of each ion species across the temperature axis (x-axis) and reduce time to quasi-steady state [4]. Unique initial configurations were generated for each simulation replicate (three replicates of NaCl brine and four replicates of seawater brine) and energetically minimised for 100 steps using a conjugate gradient and line search algorithm. An equilibration simulation was run for 1 ns at constant temperature of 40 °C and constant pressure of 1.013 25 bar. The equilibration simulation parameters were identical to those described in previous work [36], except that pressure was maintained by fluctuations in only the z-axis using Nosé–Hoover Langevin piston control under a constant area constraint. This allowed the temperature axis (x-axis) to remain fixed so as to be a consistent dimension across simulation replicates. Thermostat calibration was undertaken for each replicate of the seawater and NaCl brine solutions, identically to the methods in

previous work [36]. Thermostat target temperatures were set 20 K more extreme to achieve the desired temperature range of 20–60 °C.

The final frame of the equilibration simulation was used as the beginning of the production run. The initial velocities of replicates were randomly assigned to atoms to recreate a temperature distribution centred around 40 °C. The simulations were run for 280 ns in a constant volume and temperature environment, with both thermostats applied. The first $t_0 = 20$ ns of the production simulation was attributed as the time for the diffusing system to reach quasi-steady state, as calculated by the scaling law of [4]. Supplementary Fig. 9 shows the convergence of the NaCl brine concentration profile over the time length of the simulation, and validates $t_0 = 20$ ns as a reasonable estimate of the time to reach the quasi-steady state condition. Supplementary Fig. 10 shows the convergence of the seawater brine concentration profile over the time length of the simulation, where individual ion concentrations are considered separately. Supplementary Fig. 10a shows that $t_0 = 20$ ns is reasonable for the convergence of the ion concentration profiles that had significant initial concentrations (Cl^-, Na^+, and Mg^{2+}). Supplementary Fig. 10b and 10c assess the convergence of the seawater ions with smaller initial concentrations, Ca^{2+}, K^+, Br^-, and F^-, highlighting the sampling issue of characterising the quasi-steady state behaviour of very few ions. Overall, the quasi-steady state concentration and temperature distributions of the ions in simulation were analysed in the simulation time interval $[20, 280]$ ns.

6.5 Data analysis

All data analysis was completed using custom Fortran scripts or in Jupyter Notebooks using the packages `numpy`, `math`, `scipy.optimise`, `scipy.stats` and `pymbar`. The production portion of each simulation trajectory was used to calculate a time-averaged ion concentration, $C_{\text{ion}}(x_i)$, water concentration, $C_{\text{water}}(x_i)$, and temperature profile, $T(x_i)$, across the x-axis following the same analysis technique as described in [36], with a bin width of 1 nm (i.e. $x_i = [0.5, 1.5), [1.5, 2.5), ..., [19.5, 20.5)$ nm for $i = 0, 1, ..., 19$). We make the assumption that ions move collectively as a single species at the macro-scale level due to their electrostatic attraction, and therefore denote $C_{\text{ion}}(x_i)$ to be the combined ion concentration. The concentration profiles of individual ions were also calculated for more detailed analyses. The mass fractions of the ions were calculated from the ratio of the local mass of ions to the mean mass of the solution. Data points that lie within the thermostat regions were excluded from further analyses. A temperature range discretisation ($T \in [20, 60]$ °C with a bin width of 4.5 °C) was introduced to collect $C_{\text{ion}}(x_i)$ and $T(x_i)$ values across the simulation periodic boundary and create an average ion concentration against average temperature profile for each replicate, $C_{\text{ion}}(\overline{T})$. Note that $\overline{T}$ is the average temperature of $T(x_i)$ values collected into a given discretised temperature bin and reported with a standard deviation in the mean. $C_{\text{ion}}(\overline{T})$ was then averaged across simulation replicates (four replicates for seawater brine and three replicates for NaCl) to create $\overline{C}_{\text{ion}}(\overline{T})$. Due to the low number of simulation replicates, the spread in $\overline{C}_{\text{ion}}(\overline{T})$ was reported as a 95% confidence interval calculated from the standard deviation amongst replicates of $C_{\text{ion}}(\overline{T})$.

Thermodiffusive separation was characterised by a total concentration drop between the high ($T_{\text{hot}} = 60$ °C) and low ($T_{\text{cold}} = 20$ °C) temperature regions, evaluated from a linear fit of $\overline{C}_{\text{ion}}(\overline{T})$ for seawater brine and NaCl brine solutions. The linear fit was performed using the function `linregress` of the python `scipy.stats` package with goodness in the fit reported by an R^2 value. An error in the linear fit was characterised from the maximum difference in fits when considering the standard error in the

slope and intercept parameters, while ensuring the average ion concentration of the profile is conserved. Optimal values for the binary ion–water Soret coefficient, S_T, for seawater brine and NaCl brine were found from the linear estimates of the ion concentration drop in each solution, using the golden-section method described in Supplementary Method 5.

6.6 Thermodiffusive behaviour of individual ions in a seawater substitute

MD simulations can provide insight into the ion–ion interactions in a multi-component solution. Here we decompose the behaviour of the modelled seawater brine solution into the individual ion concentration profiles to better understand why the seawater brine exhibited stronger thermodiffusive separation than NaCl brine.

Supplementary Fig. 11 compares the concentration profiles of individual ions in NaCl brine and seawater brine. We focus on the ion species with significant concentrations in the initial brine solutions, that is, Cl^-, Na^+ and Mg^{2+}. The concentration profiles of the other ion species in the modelled seawater brine, Ca^{2+}, K^+, F^- and Br^-, are disregarded due to the statistical sampling issue of the small number of ions in the model solution. In Supplementary Fig. 11, each ion concentration profile is normalised to the average concentration of that ion in the solution, to allow comparison of the strength of ion separation. All ions showed thermophobic separation, with concentration increasing as temperature decreases. The Cl^- and Na^+ profiles are well described by linear lines of best fit. The Mg^{2+} profile is also reasonably described by a linear line of best fit, however close to 25 °C the Mg^{2+} profile flattens, showing evidence of the direction of thermodiffusion switching (the inversion temperature). The strength of separation for each ion species is characterised by a separation percentage, that is the difference between the normalised ion concentration at the temperature extremes, $T_{\text{cold}} = 20\,°C$ and $T_{\text{hot}} = 60\,°C$, as given by the linear fit. Supplementary Fig. 11a, compares the Cl^- and Na^+ concentration profiles in NaCl brine. Supplementary Fig. 11b compares the Cl^-, Na^+, and Mg^{2+} concentration profiles in modelled seawater brine.

Approximating an aqueous electrolyte solution as a binary solution of ions and water is appropriate for single salt solutions, but is a simplification of the ion–ion interactions within multi-component electrolyte solutions. From Supplementary Fig. 11a we see that the Cl^- and Na^+ concentration profiles are identical to one-another, with a separation percentage of 5.4%. In this single salt solution, the quasi-steady state dynamics of the cation and anion are coupled via electrostatic attraction, and so the system can reasonably be approximated as a binary solution of ions and water. In a multi-component electrolyte, such as our modelled seawater brine, this is not the case. From Supplementary Fig. 11b we see that the Cl^-, Na^+ and Mg^{2+} concentration profiles differ from one-another. Mg^{2+} has the largest separation, characterised by a separation percentage of 25.4%, followed by Cl^- with a separation percentage of 10.6%, and then Na^+ with a separation percentage of 6.7%. The quasi-steady state dynamics of these cations and anion are influenced by their electrostatic interactions, however each ion maintains a unique concentration profile. Making the approximation that a multi-component electrolyte solution can be reduced to a binary system of ions and water is an over simplification that removes detail of the ion–ion interactions. It is interesting to note, therefore, that thermodiffusive desalination of natural seawater may remove certain ions from the solution faster and more successfully than other ions.

A key result from this work is that the salinity reduction of a multi-ion seawater substitute via TDD would be comparable to or larger than that predicted for NaCl/H_2O solutions. In particular, the MD simulations predict that the modelled seawater brine has an effective Soret coefficient ca. 1.8 times larger than that of NaCl brine. By analysing the individual ion components of the seawater brine, show in Supplementary Fig. 11b, we propose that the stronger thermodiffusive separation predicted for the modelled seawater brine is likely driven by the strong separation of the Mg^{2+} ions and consequent 'flow-on' electrostatic interaction between Mg^{2+} and the other ions in solution. Mg^{2+} has the strongest individual ion separation of 25.4%. Cl^-, an anion, has a primary response to the non-uniform Mg^{2+} concentration due to electrostatic attraction, and consequently the Cl^- separation percentage is increased to 10.6% (larger than the 5.4% separation of Cl^- in NaCl brine). Na^+, a monovalent cation, responds to the higher Cl^- separation (and consequently has a secondary response to the large Mg^{2+} separation), increasing the Na^+ separation percentage to 6.7%. Note that both the Cl^- and Na^+ separations in the presence of Mg^{2+} are increased from the 5.4% separation in the NaCl brine, as in Supplementary Fig. 11a. Overall, we propose that the strong separation of the Mg^{2+} ions and consequent 'flow-on' electrostatic interaction between Mg^{2+} and the other ions in solution is the likely driver of the larger effective Soret coefficient predicted for the modelled seawater brine.

Supplementary Method 7: Burgers cascade modelling

The following flow assumptions are made in the modelling and design optimisation of the Burgers cascade: (1) mixing of the two streams happens instantaneously at the inlet of each cell, (2) the concentration profile fully develops in each cell, and (3) the two streams at the outlet of the cell are perfectly bifurcated at the horizontal mid-plane. Regarding the operating temperature, thermodiffusion is a process that does not involve any phase change so the maximum temperature should be less than 100 °C under sea-level pressure of 1 atm to avoid boiling. For TDD to be competitive with existing and emerging desalination technologies (e.g. see Supplementary Table 3), it is crucial that we utilise low-grade thermal energy such as industrial waste heat or solar thermal energy for heating, while the cooler environment acts as a heat sink *via* natural convection. Thus it is reasonable to assume the cold side of the Burgers cascade is at $T_{\mathrm{cold}} = 30\,°\mathrm{C}$ and the hot side is at $T_{\mathrm{hot}} = 90\,°\mathrm{C}$. The freestream water temperature for the heat sink could be less than $T_{\mathrm{sur}} = 10\,°\mathrm{C}$ if sourced from beneath the oceanic mixed layer. Given that the convective heat transfer coefficient h_{conv} for water under natural convection is generally less than $1000\,\mathrm{W\,m^{-2}\,K^{-1}}$ [40], maximum convective heat flux $q_{\mathrm{conv,\,max}} = h_{\mathrm{conv}}\Delta T_{\mathrm{conv}} = 1000\,\mathrm{W\,m^{-2}\,K^{-1}} \times 20\,\mathrm{K}$ is less than $20\,\mathrm{kW\,m^{-2}}$. Note that the heat flux of $20\,\mathrm{kW\,m^{-2}}$ is larger than one sun at normal irradiance (ca. $1\,\mathrm{kW\,m^{-2}}$). A simple solar collector is enough to provide a concentration ratio of 20 to achieve the required heat fluxes when supplied *via* solar thermal energy. Next, based on Fourier's law of heat conduction, $q = k\frac{\Delta T}{h} < q_{\mathrm{conv,\,max}}$ informs that the channel height h should be greater than 1.9 mm.

The next parameter to consider is the volumetric flow rate Q. Based on the assumption that the concentration profile has fully developed in each cell, the residence time of the fluid in each cell is equivalent to τ_{th}, which is proportional to h^2 as discussed in the Supplementary Method 1. In addition, the number of cells required in the Burgers cascade is determined by the target salinity drop, e.g. for a salinity drop of 25 000 ppm, $M = 185$ and $N = 20$ is required. Once we impose another limit, the total surface area A of the Burgers cascade (planar side) and the lateral dimensions of each individual cell, width w and length l, are interdependent as follows: $lw = \frac{A}{MN}$. The total flow rate then becomes $Q = \frac{l}{\tau_{\mathrm{th}}}whN = \frac{Ah}{M\tau_{\mathrm{th}}}$. To conclude the analysis, for a fixed condition of temperature and area, the yield of the Burgers cascade for certain target low-concentration yield is only dependent on the cell height h. An example is shown in Supplementary Fig. 13.

Supplementary Method 8: Energy consumption and thermodynamic limit

Another important aspect to consider is the energy consumption. The energy consumption of the single-channel TDU is broken into two parts, one is the thermal energy, which is around 232 W (with a $23.2\,\mathrm{kW\,m^{-2}}$ heat flux) and the other is the work rate $\dot{W} = 1.4\,\mu\mathrm{W}$ to overcome a pressure drop of $\Delta P = 17\,\mathrm{Pa}$. Here, we apply a simple analytical solution to estimate the energy consumption, because the high degree of agreement between the CFD results and the experimental results implies that the TDU can be modelled as a two-dimensional channel. We consider a one-dimensional flow pattern (only dependent on y; Supplementary Fig. 2) in the channel, whose vertical projection is taken as the calculation domain. Thus the energy equation can be expressed as:

$$\frac{D}{Dt}\int_{\text{sys}} e\rho \, \mathrm{d}\forall = \left(\sum \dot{Q} + \sum \dot{W}\right)_{\text{sys}}. \tag{24}$$

Here, the time rate of increase of the total stored energy of the system is equivalent to the rate of net heat transfer $\dot{Q}$ and work transfer $\dot{W}$ in to the system, and e is the stored energy per unit mass. For a simplified assumption where the flow velocity is constant and there is no height difference between the inlet and the outlet of the channel, the stored energy can be simplified to be the internal energy so that $e = \check{u}$ and the left-hand side of the equation can be simplified. TDD is a single-phase thermal process and, in the experiments, ΔT across the channel was established by two water baths. The heat flux per unit area was $q = k\frac{\Delta T}{h} = 23.2\,\mathrm{kW\,m^{-2}}$ and $\dot{Q}_{\text{in}} = 232\,\mathrm{W}$ maintains a temperature difference ΔT of $37\,\mathrm{K}$. However, ΔT can be established through solar irradiation and advection and such methods do no require any electrical energy input, so the thermal energy transport $\dot{Q}$ may be excluded from the energy consumption calculation. Such exclusion is reasonable given that moderate-temperature heat is rarely used for practical applications. $\dot{W}$ is only related to the fluid pressure acting on the control surface, and in the case of the TDU:

$$\dot{W} = \int_{\text{cs}} -P\mathbf{u}\hat{n}\,\mathrm{d}A = Q(-P_{\text{in}} + P_{\text{out}}), \tag{25}$$

where Q is the volumetric flow rate. Therefore, the only electrical energy input into the TDU channel is related to operation of the pumps to overcome the pressure drop. For a rectangular channel, the pressure drop ΔP can be calculated as:

$$\Delta P = f\frac{l}{D_{\text{h}}}\frac{\rho V^2}{2}, \tag{26}$$

where f is the friction factor and for a rectangular cross section with an aspect ratio of 0.05, $f = \frac{C}{\text{Re}} = 7.35$. Hydrodynamic diameter D_{h} is calculated as $D_{\text{h}} = \frac{2ab}{a+b} = 0.0019\,\mathrm{m}$. Thus $\Delta P = 17\,\mathrm{Pa}$. Based on Eq. (25), the work transfer becomes $\dot{W} = 1.4\,\mu\mathrm{W}$.

As discussed in the the Supplementary Method 7, for a target yield concentration, there exits a minimum value of M and N that allows the Burgers cascade to reach the target concentration. Under this condition, once the lateral area A of the Burgers cascade is fixed, then ΔP in each individual cell can be calculated in the same way as the single channel TDU, with varying cell dimensions h, w, and l (w and l are interdependent). The total pressure can be calculated as $\Delta P \times M \times N$ when ignoring the impedance imposed by the bifurcation and recombination structure at the ends of each cell. To verify the analytical solution, we compare it to a high-fidelity CFD that was performed for a Burgers cascade in the literature [41]. For the Burgers cascade that is made of several of these mini parallel-plate channels, the ΔP is 7.9 Pa in their paper. Following the analytical solution and multiplying by the number of cells in the CFD model, we obtain $\Delta P = 6.5\,\mathrm{Pa}$ for the CO_2/H_2O gas mixture. Therefore, the analytical solution provides a good approximation for the pressure drop in the channel. Moreover, the analytical solution can be scaled to approximate the ΔP not only in a parallel-plate TDU channel, but also in a Burgers cascade that contains many small cells. This is how the pressure drop is calculated in

Supplementary Fig. 13a. We noticed that for a target yield concentration of 5000 ppm, typical pressure drop does not exceed 10 kPa with a flow rate around 10 L day^{-1}. Based on Eq. (25), the electrical power consumption for such setup is 1.2 mW and the electrical energy consumption to desalinated water to this salinity is 3 $\mathrm{Wh_e\,m^{-3}}$. Importantly, we excluded the thermal energy from the energy consumption and only focus on the electrical energy required to drive the fluid flow. Furthermore, note that the pump efficiency was not considered in this analysis because a suitable pump selection may produce efficiencies above 90%.

The well-known thermodynamic limit (i.e. theoretical minimum energy of separation) for desalination is around 1 kWh m^{-3} based on the Gibbs free energy for separation [5]. However, the thermodynamic limit is dependent on the the concentration of feedwater and yield water, as well as the recovery rate R_w of the desalination process. The minimal specific energy consumption per unit volume of produced water $\mathrm{SEC_{min}}$ can be calculated as:

$$\mathrm{SEC_{min}} = 2RT\left[\frac{c_\mathrm{f}}{R_\mathrm{w}}\ln\frac{c_\mathrm{b}}{c_\mathrm{f}} - c_\mathrm{y}\ln\frac{c_\mathrm{b}}{c_\mathrm{y}}\right], \tag{27}$$

where c is the molar concentration and subscript f, y, b denotes feedwater, yield water and the brine water, respectively. R is the gas constant and T is the absolute temperature. When c_f and c_y are known, then $c_\mathrm{b} = \frac{c_\mathrm{f}-R_\mathrm{w}c_\mathrm{y}}{1-R_\mathrm{w}}$ based on mass conservation. The above equation can be further simplified such that $\mathrm{SEC_{min}}$ is only dependent on the recovery rate:

$$\mathrm{SEC_{min}} = 2RT\left[\frac{c_\mathrm{f}}{R_\mathrm{w}}\ln\frac{c_\mathrm{f}-R_\mathrm{w}c_\mathrm{y}}{c_\mathrm{f}(1-R_\mathrm{w})} - c_\mathrm{y}\ln\frac{c_\mathrm{f}-R_\mathrm{w}c_\mathrm{y}}{c_\mathrm{y}(1-R_\mathrm{w})}\right]. \tag{28}$$

As shown in Supplementary Fig. 14a, desalinating from 35 000 ppm to 0 ppm at a recovery rate of 50% yields a theoretical minimum energy of separation of $\mathrm{SEC_{min}} = 1.1\,\mathrm{kWh\,m^{-3}}$. While in Supplementary Fig. 14b, desalinating from 30 000 ppm to 5000 ppm at a recovery rate of 10% requires $\mathrm{SEC_{min}} = 0.4\,\mathrm{kWh\,m^{-3}}$. In contrast, electrical energy alone consumed by TDD Burgers cascade is a small fraction of the $\mathrm{SEC_{min}}$.

Electric power savings when using TDD as a pre-treatment desalination method

When considering the potential of TDD as a pre-treatment method for other desalination technologies that are more energy efficient when the feedwater salinity is lower, the specific energy consumption per unit volume of produced fresh water for each technology should be calculated. For example, in the case of RO, $\mathrm{SEC_{RO}}$ is directly proportional to the applied hydraulic pressure [42], which scales almost linearly with the feedwater salinity. While using the same model as in [42], we compared the power consumption between two desalination pathways: (A) using RO as the sole method to desalinate from seawater concentration to an acceptable potable water standard, i.e. from 30 000 ppm to 1000 ppm; and (B) using TDD first as a pre-treatment method to drop seawater concentration to 5000 ppm and then RO to further desalinate to 1000 ppm. We found that the electric power consumption is significantly reduced when opting for pathway B (hybrid TDD–RO) instead of pathway A (standalone RO), with a reduction in electric power from 4.5 $\mathrm{kWh_e\,m^{-3}}$ (A) to 0.7 $\mathrm{kWh_e\,m^{-3}}$ (B), which represents electrical

energy savings of over 80%. It is also noted that when the feedwater salinity is lower than 5000 ppm and the target salinity is 1000 ppm, ED is more energy-efficient than RO [42]. Hence, a hybrid TDD–ED approach could be another feasible combination with electrical energy savings expected to exceed 80%.

Supplementary Table 4 | Reference list for desalination technology comparison (used in Fig. 6 main manuscript). The literature source for the different developed desalination technologies. The different technologies are: reverse osmosis (RO), multi-stage flash (MSF) and multi-effect distillation (MED), eletrodialysis (ED), Adsorption, capacitive deionization (CDI), novel solar-driven (NSD) desalination, e.g. interfacial evaporation and contactless steam generation, and membrane distillation (MD). The thermodiffusive desalination (TDD) method developed in this work is compared against these technologies.

Desalination technology	Label on Fig. 6	Ref.
RO	1	Kucera, 2023 [43]
	2, 3	Karabelas et al., 2018 [44]
	4	Patel, Biesheuvel and Elimelech, 2021 [42]
	5	World Bank, 2019 [45]
MSF and MED	6, 7	World Bank, 2019 [45]
ED	8, 9	Patel, Biesheuvel and Elimelech, 2021 [42]
	10	Doornbusch et al., 2021 [46]
	11	Turek, 2003 [47]
Adsorption	12	Yang et al., 2013 [48]
	13, 14	Wibowo et al., 2017 [10]
	15, 16	Paul, Dynes and Chang, 2017 [49]
	17	Mishra and Ramaprabhu, 2011 [50]
	18	Ou et al., 2020 [9]
CDI	19	Roshan et al., 2022 [51]
	20	Hsu et al., 2020 [52]
	21	Liu et al., 2015 [13]
	22	Wang et al., 2019 [53]
	23–25	Lado et al., 2015 [54]
	26	Porada et al., 2012 [12]
NSD	27	Cooper et al., 2018 [55]
	28	Gong et al., 2021 [17]
	29	Ni et al., 2018 [21]
	30	He et al., 2019 [56]
	31	Dongare et al., 2019 [57]
	32	Ni et al., 2016 [58]
	33	Li et al., 2021 [19]
MD	34	Ali et al., 2012 [59]
	35	Jantaporn, Ali and Aimar, 2017 [60]
	36	Bouguecha, Hamrouni and Dhahbi, 2005 [61]

Supplementary References

1. Torres, J. F., Komiya, A., Henry, D. & Maruyama, S. Measurement of Soret and Fickian diffusion coefficients by orthogonal phase-shifting interferometry and its application to protein aqueous solutions. *Journal of Chemical Physics* **139,** 23968083 (2013).
2. Mialdun, A. & Shevtsova, V. Measurement of the Soret and diffusion coefficients for benchmark binary mixtures by means of digital interferometry. *Journal of Chemical Physics* **134,** 044524 (2011).
3. Torres, J. F., Komiya, A., Shoji, E., Okajima, J. & Maruyama, S. Development of phase-shifting interferometry for measurement of isothermal diffusion coefficients in binary solutions. *Optics and Lasers in Engineering* **50,** 1287–1296 (2012).
4. Diaz-Marquez, A. & Stirnemann, G. In silico all-atom approach to thermodiffusion in dilute aqueous solutions. *The Journal of Chemical Physics* **155,** 174503 (2021).
5. Wang, L., Violet, C., Duchanois, R. M. & Elimelech, M. Derivation of the Theoretical Minimum Energy of Separation of Desalination Processes. *Journal of Chemical Education* **97,** 4361–4369 (2020).
6. Cao Q. Pui, D. Y. H. & Lipiński, W. A concept of a novel solar-assisted large-scale cleaning system (SALSCS) for urban air remediation. *Aerosol Air Quality Research* **15,** 1–10 (2015).
7. Sengupta, A., Li, Z., Song, L. F., Li, P. & Merz, K. M. Parameterization of Monovalent Ions for the OPC3, OPC, TIP3P-FB, and TIP4P-FB Water Models. *Journal of Chemical Information and Modeling* **61,** 869–880 (2021).
8. Li, Z., Song, L. F., Li, P. & Merz, K. M. Systematic Parametrization of Divalent Metal Ions for the OPC3, OPC, TIP3P-FB, and TIP4P-FB Water Models. *Journal of Chemical Theory and Computation* **16,** 4429–4442 (2020).
9. Ou, R. *et al.* A sunlight-responsive metal–organic framework system for sustainable water desalination. *Nature Sustainability* **3,** 1052–1058 (2020).
10. Wibowo, E., Rokhmat, M., Sutisna, Khairurrijal & Abdullah, M. Reduction of seawater salinity by natural zeolite (Clinoptilolite): Adsorption isotherms, thermodynamics and kinetics. *Desalination* **409,** 146–156 (2017).
11. Gibb, N. P., Dynes, J. J. & Chang, W. Synergistic desalination of potash brine-impacted groundwater using a dual adsorbent. *Science of the Total Environment* **593-594,** 99–108 (2017).
12. Porada, S., Sales, B. B., Hamelers, H. V. & Biesheuvel, P. M. Water desalination with wires. *Journal of Physical Chemistry Letters* **3,** 1613–1618 (2012).
13. Liu, Y. *et al.* Nitrogen-doped porous carbon spheres for highly efficient capacitive deionization. *Electrochimica Acta* **158,** 403–409 (2015).
14. Zhang, Y., Ji, L., Zheng, Y., Liu, H. & Xu, X. Nanopatterned metal–organic framework electrodes with improved capacitive deionization properties for highly efficient water desalination. *Separation and Purification Technology* **234,** 116124 (2020).
15. Forrestal, C., Xu, P. & Ren, Z. Sustainable desalination using a microbial capacitive desalination cell. *Energy and Environmental Science* **5,** 7161–7167 (2012).
16. Kim, S. J., Ko, S. H., Kang, K. H. & Han, J. Direct seawater desalination by ion concentration polarization. *Nature Nanotechnology* **5,** 297–301 (2010).
17. Gong, B. *et al.* Multifunctional solar bamboo straw: Multiscale 3D membrane for self-sustained solar-thermal water desalination and purification and thermoelectric waste heat recovery and storage. *Carbon* **171,** 359–367 (2021).
18. Zhang, L. *et al.* Highly efficient and salt rejecting solar evaporation via a wick-free confined water layer. *Nature Communications* **13,** 35165279 (2022).
19. Li, D. *et al.* A flexible and salt-rejecting electrospun film-based solar evaporator for economic, stable and efficient solar desalination and wastewater treatment. *Chemosphere* **267,** 33213877 (2021).
20. Tao, P. *et al.* Solar-driven interfacial evaporation. *Nature Energy* **3,** 1031–1041 (2018).

21. Ni, G. *et al.* A salt-rejecting floating solar still for low-cost desalination. *Energy and Environmental Science* **11,** 1510–1519 (2018).

22. Mao, Y. *et al.* Roughness-enhanced hydrophobic graphene oxide membrane for water desalination via membrane distillation. *Journal of Membrane Science* **611,** 118364 (2020).

23. Huang, Y. X., Wang, Z., Jin, J. & Lin, S. Novel Janus Membrane for Membrane Distillation with Simultaneous Fouling and Wetting Resistance. *Environmental Science and Technology* **51,** 13304–13310 (2017).

24. Wang, Y., Liu, X., Ge, J., Li, J. & Jin, Y. Distillation performance in a novel minichannel membrane distillation device. *Chemical Engineering Journal* **462,** 142335 (2023).

25. Lu, K. J., Cheng, Z. L., Chang, J., Luo, L. & Chung, T. S. Design of zero liquid discharge desalination (ZLDD) systems consisting of freeze desalination, membrane distillation, and crystallization powered by green energies. *Desalination* **458,** 66–75 (2019).

26. Xu, S., Komiya, A., Corry, B. & Torres, J. F. Scaling up thermodiffusive separation through a microchannel. *Proceedings of the 23rd Australasian Fluid Mechanics Conference,* 438 (2022).

27. Caldwell, D. R. Thermal and Fickian diffusion of sodium chloride in a solution of oceanic concentration. *Deep-Sea Research and Oceanographic Abstracts* **20,** 1029–1039 (1973).

28. Patankar, S. *Numerical Heat Transfer and Fluid Flow* (Hemishpere Publising Cooperation, 1980).

29. Vigolo, D., Rusconi, R., Stone, H. A. & Piazza, R. Thermophoresis: Microfluidics characterization and separation. *Soft Matter* **6,** 3489–3493 (2010).

30. Potter, M. C., Wiggert, D. C. & Ramadan, B. H. *Mechanics of Fluids* 5th edition (Cengage Learning, 2016).

31. Huber, D., Oskooei, A., Casadevall Solvas, X., Andrew Demello & Kaigala, G. V. Hydrodynamics in Cell Studies. *Chemical Reviews* **118,** 2042–2079 (2018).

32. Vanderplaats, G. N. *Numerical Optimization Techniques for Engineering Design: with Applications* 41–49 (McGraw-Hill College, 1984).

33. Blanco, P. *et al.* Thermodiffusion coefficients of binary and ternary hydrocarbon mixtures. *Journal of Chemical Physics* **132,** 20331304 (2010).

34. Gebhardt, M., Köhler, W., Mialdun, A., Yasnou, V. & Shevtsova, V. Diffusion, thermal diffusion, and Soret coefficients and optical contrast factors of the binary mixtures of dodecane, isobutylbenzene, and 1,2,3,4-tetrahydronaphthalene. *Journal of Chemical Physics* **138,** 114503 (2013).

35. Phillips, J. C. *et al.* Scalable molecular dynamics on CPU and GPU architectures with NAMD. *Journal of Chemical Physics* **153,** 32752662 (2020).

36. Hutchinson, A., Torres, J. & Corry, B. Modeling thermodiffusion in aqueous sodium chloride solutions — Which water model is best? *The Journal of Chemical Physics* **156,** 164503 (2022).

37. Wang, L. P., Martinez, T. J. & Pande, V. S. Building force fields: An automatic, systematic, and reproducible approach. *Journal of Physical Chemistry Letters* **5,** 1885–1891 (2014).

38. Belkin, M., Chao, S.-H., Giannetti, G. & Aksimentiev, A. Modelling thermophoretic effects in solid-state nanopores. *Journal of Computational Electronics* **23,** 1–7 (2014).

39. Martínez, L., Andrade, R., Birgin, E. G. & Martínez, J. M. Packmol: A package for building initial configurations for molecular dynamics simulations. *Journal of Computational Chemistry* **13,** 2157–2164 (2009).

40. Bergman, T. L., Lavine, A. S., Incropera, F. P. & Dewitt, D. P. *Introduction to Heat Transfer* 6th ed. (John Wiley and Sons, Inc., 2011).

41. Kyoda, T., Saiki, T., Matsumoto, S., Watanabe, S. & Ono, N. Performance improvement of a micro-structured gas separator utilizing the Soret effect. *Journal of Thermal Science and Technology* **17,** 1–15 (2022).

42. Patel, S. K., Biesheuvel, P. M. & Elimelech, M. Energy Consumption of Brackish Water Desalination: Identifying the Sweet Spots for Electrodialysis and Reverse Osmosis. *ACS ES&T Engineering* **1,** 851–864 (2021).

43. Kucera, J. *Reverse Osmosis: Design, Processes, and Applications for Engineers* (Scrivener Publishing and John Wiley & Sons, Inc, 2010).
44. Karabelas, A. J., Koutsou, C. P., Kostoglou, M. & Sioutopoulos, D. C. Analysis of specific energy consumption in reverse osmosis desalination processes. *Desalination* **431,** 15–21 (2018).
45. *The Role of Desalination in an Increasingly Water-Scarce World* (World Bank, 2019).
46. Doornbusch, G. *et al.* Multistage electrodialysis for desalination of natural seawater. *Desalination* **505,** 114973 (2021).
47. Turek, M. Cost effective electrodialytic seawater desalination. *Desalination* **153,** 371–376 (2003).
48. Yang, H. Y. *et al.* Carbon nanotube membranes with ultrahigh specific adsorption capacity for water desalination and purification. *Nature Communications* **4,** 23941894 (2013).
49. Paul, B., Dynes, J. J. & Chang, W. Modified zeolite adsorbents for the remediation of potash brine-impacted groundwater: Built-in dual functions for desalination and pH neutralization. *Desalination* **419,** 141–151 (2017).
50. Mishra, A. K. & Ramaprabhu, S. Functionalized graphene sheets for arsenic removal and desalination of sea water. *Desalination* **282,** 39–45 (2011).
51. Roshan, B., Rasoulzadeh, H., Massoudinejad, M., Saadani, M. & Sanaei, D. Enhanced desalination efficiency of flow-through capacitive deionization cell by mesh electrode with granular aerogel carbon in the removal of ions from synthetic and real samples. *Water Reuse* **12,** 33–51 (2022).
52. Hsu, C. C., Tu, Y. H., Yang, Y. H., Wang, J. A. & Hu, C. C. Improved performance and long-term stability of activated carbon doped with nitrogen for capacitive deionization. *Desalination* **481,** 114362 (2020).
53. Wang, Z. *et al.* Nanoarchitectured metal-organic framework/polypyrrole hybrids for brackish water desalination using capacitive deionization. *Materials Horizons* **6,** 1433–1437 (2019).
54. Lado, J. J. *et al.* Continuous cycling of an asymmetric capacitive deionization system: An evaluation of the electrode performance and stability. *Journal of Environmental Chemical Engineering* **3,** 2358–2367 (2015).
55. Cooper, T. A. *et al.* Contactless steam generation and superheating under one sun illumination. *Nature Communications* **9,** 1–10 (2018).
56. He, S. *et al.* Nature-inspired salt resistant bimodal porous solar evaporator for efficient and stable water desalination. *Energy and Environmental Science* **12,** 1558–1567 (2019).
57. Dongare, P. D., Alabastri, A., Neumann, O., Nordlander, P. & Halas, N. J. Solar thermal desalination as a nonlinear optical process. *Proceedings of the National Academy of Sciences of the United States of America* **116,** 13182–13187 (2019).
58. Ni, G. *et al.* Steam generation under one sun enabled by a floating structure with thermal concentration. *Nature Energy* **1,** 1–8 (2016).
59. Ali, M. I., Summers, E. K., Arafat, H. A. & Lienhard V, J. H. Effects of membrane properties on water production cost in small scale membrane distillation systems. *Desalination* **306,** 60–71 (2012).
60. Jantaporn, W., Ali, A. & Aimar, P. Specific energy requirement of direct contact membrane distillation. *Chemical Engineering Research and Design* **128,** 15–26 (2017).
61. Bouguecha, S., Hamrouni, B. & Dhahbi, M. Small scale desalination pilots powered by renewable energy sources: Case studies. *Desalination* **183,** 151–165 (2005).
